# Environmental Factors and Co‐Occurrence Patterns Influence Dorsal Brightness in Two Jumping Mice Species in the Western United States

**DOI:** 10.1002/ece3.73119

**Published:** 2026-02-22

**Authors:** José Gabriel Martínez‐Fonseca, Erin P. Westeen, Jennifer L. Zahratka, Carol L. Chambers

**Affiliations:** ^1^ School of Forestry Northern Arizona University Flagstaff Arizona USA; ^2^ Department of Environmental Science, Policy, and Management University of California Berkeley California USA; ^3^ Museum of Vertebrate Zoology University of California Berkeley California USA; ^4^ School of Life Sciences Arizona State University Tempe Arizona USA; ^5^ Biological Resources Durango Colorado USA

## Abstract

The phenotype is the result of interactions between an organism's genotype and its environment. One phenotypic trait of interest is dorsal coloration, which animals use to communicate, avoid detection from predators, thermoregulate, and more. Here we used standardized photography of museum specimens to explore the variation in dorsal brightness of the endangered New Mexico jumping mouse ( *Zapus luteus* ) in areas of allopatry and sympatry with the western jumping mouse ( *Z. princeps* ) in the southwestern United States. For *Z. luteus* we found statistically significant differences in brightness among isolated, genetically differentiated populations in Arizona and New Mexico. We additionally found significant, predictable interspecific differences in color between *Z. luteus* and *Z. princeps* where they co‐occur; however, these differences would be difficult to distinguish visually. We also found evidence that the dorsal brightness of both species is associated with geographic (elevation, latitude) and bioclimatic (precipitation, temperature) features, as well as interspecific interactions (syntopy patterns). Our findings emphasize that both biotic and abiotic factors influence dorsal coloration in free‐living organisms, and highlight the importance of using multiple lines of evidence to identify co‐occurring congeners in the field. For endangered species like *Z. luteus* , correct identification can have important management implications.

## Graphical abstract

We examined how dorsal coloration varies in the endangered New Mexico jumping mouse ( *Zapus luteus* ) and its close relative, the western jumping mouse (*Z. princeps*) in relation to environmental variables and co‐occurence patterns using standardized photographs of museum specimens. We found that dorsal brightness varied significantly among genetically distinct populations and was influenced by geographic, climatic, and interspecific factors. These results highlight that both environmental and biological interactions drive coloration differences and stress the need for accurate species identification to guide conservation efforts.

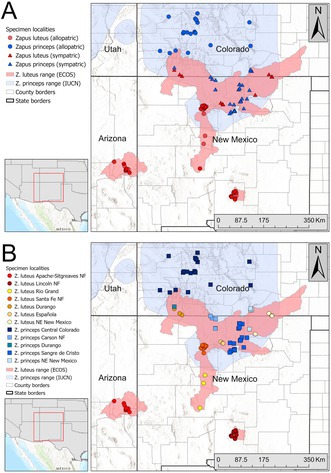

## 1 Introduction

Natural selection acts on phenotypes—the set of observable traits in an organism—and in turn, the phenotype is the result of interactions between the organismal genotype and environment (Peaston and Whitelaw 2006). Studies of intraspecific phenotypic variation are fundamental to ecology and evolution because persistent phenotypic differences suggest differential selection. One such phenotypic trait, coloration, is of particular interest because animals use color to communicate both intra‐ and interspecifically, and for functions such as thermoregulation, predator avoidance, prey capture, and mate attraction (Stuart‐Fox et al. 2017; Duarte et al. 2025).

In a pattern well documented across insects, reptiles, and mammals, temperature and moisture conditions can influence animal coloration (Clusella Trullas et al. 2007; Zverev et al. 2018; Delhey et al. 2019; Martínez‐Freiría et al. 2020). The pattern of darker coloration in cold climates is known as Bogert's rule or thermal melanism in reference to melanin, the pigment that provides color to skin and hair across vertebrate animals (Quevedo Jr. and Holstein 2006; Caro and Mallarino 2020). Darker dorsal coloration can help organisms absorb more solar radiation and more effectively thermoregulate in colder environments (Clusella Trullas et al. 2007). Thermal melanism is often observed along elevational and latitudinal gradients, where populations occurring at higher elevations and latitudes reveal darker coloration (Alho et al. 2010). Another biogeographic pattern that associates coloration with climate is Gloger's rule, which suggests that animals should be darker in warmer and wetter climates; this pattern has also been observed in mammals across tropical and temperate regions (Delhey et al. 2019; Cerezer et al. 2024). Melanism can provide better concealment in wetter environments when compared to those living in drier landscapes (Goldenberg et al. 2024). However, temperature and precipitation can have asymmetric effects across populations of the same species (Mader et al. 2022).

Changes in melanin‐based coloration are also observed in relation to substrate matching or predator camouflaging (Lillywhite et al. 1977). For example, rock pocket mice ( *Chaetodipus intermedius* ) are usually light colored, but populations can be dark (melanic) in areas with lava flows (Nachman et al. 2003). Color patterns can also be influenced by interactions with other species, including competitors (Cerezer et al. 2024). The similarity in requirements between closely related species results in increased competition, and where they occur in sympatry, divergence of phenotypic characters can offset competition (Brown and Wilson 1956; Martin et al. 2015; Montenegro et al. 2019; Westeen et al. 2023). Thus, color differences may be more pronounced in sympatry if competition drives differences in habitat use or thermal resources. Alternatively, if the environment imposes sufficient selective pressure, habitat filtering for species with shared phenotypic traits may occur. Examining the distribution of phenotypes between closely related species in sympatry and allopatry can illuminate the roles of interspecific interactions and the environment on patterns of phenotypic variation.

Quantifying color into its constituent components such as hue (the dominant wavelength), saturation (the purity or intensity), and value (a measure of brightness) can allow for the examination of these components in relation to biological function (Duarte et al. 2025). Brightness in particular is relevant to crypsis or camouflage because many predators use the differences in brightness to detect prey (Stuart‐Fox et al. 2017). Cryptic coloration is especially important for animals that lack other anti‐predator defenses, and many notable examples of color matching come from rodents (Nachman et al. 2003; Cerezer et al. 2024).

**FIGURE 1:**
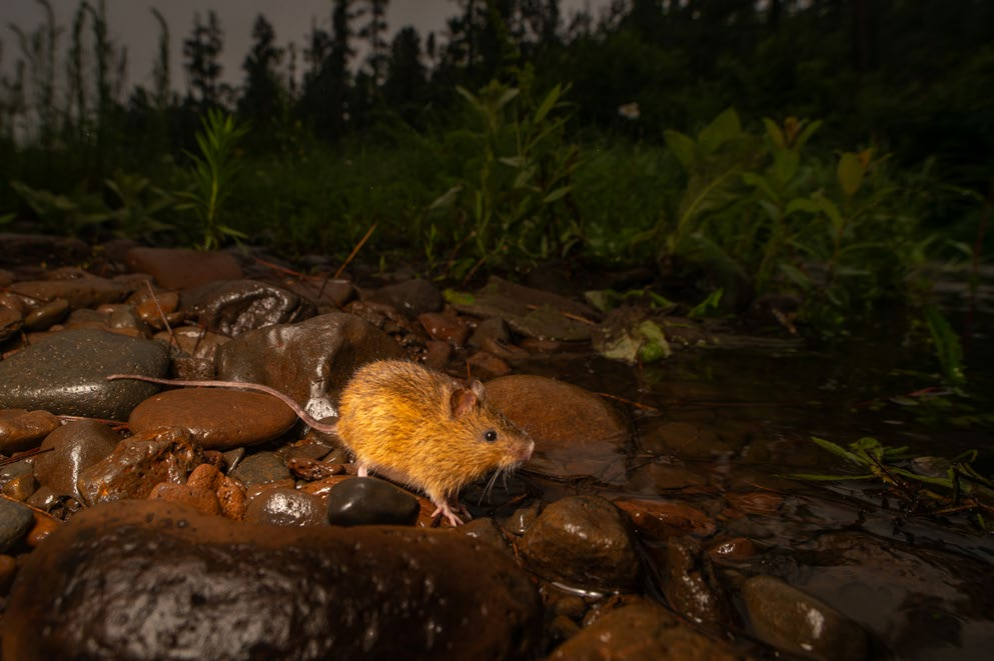
New Mexico jumping mouse ( *Zapus luteus* ) moving across its native riparian habitat in the White Mountains (Apache and Navajo Counties) of eastern Arizona.

Small mammals inhabiting heterogeneous landscapes provide a powerful opportunity to examine how environmental filtering and interspecific interactions shape phenotypic variation at fine spatial scales. North America is home of 9 species of jumping mice (*Zapus* spp.; family Zapodidae), of which three occur in Colorado, two in New Mexico, and one in Arizona (Malaney et al. 2017). The species endemic to the Southwest, the New Mexico jumping mouse ( *Z. luteus* ; Figure 1) was formerly considered a subspecies of *Z. hudsonius* and is listed under the Endangered Species Act (USFWS 2014, 2023; Malaney et al. 2017). A second species, the western jumping mouse ( *Z. princeps* ) has a greater distribution across the United States and Canada with at least 11 recognized subspecies (Blake Hart et al. 2004). Both species of jumping mice, *Z. luteus* and *Z. princeps* , are sympatric and often syntopic in southwestern Colorado and some portions of northern New Mexico. Both species can be found in riparian areas although *Z. princeps* also uses areas away from water sources that retain high soil moisture and diverse herbaceous vegetation (Blake Hart et al. 2004).

For the endangered status of *Z. luteus* , correct identification of the species throughout its range has important management implications (USFWS 2023). However, field identification of these similar‐looking species is currently based primarily on geographic location and elevation, with no widely accepted morphological markers for live animal identification. This results in great difficulty for land managers and researchers to identify species in sites where the two species overlap. Differences in dorsal coloration across populations and between co‐occurring species could be related to geographic features such as elevation or latitude, predation pressure, or competition leading to niche partitioning in areas of sympatry. Furthermore, differences in phenotypic variation can also be an indication of genetic diversity, which could be of special importance in conservation efforts for *Z. luteus* (Malaney et al. 2017; USFWS 2020, 2023).

Here, we quantified coloration of the dorsal pelage of two species in the genus *Zapus* to assess the relative roles of environmental pressures on phenotypic expression within and between species. We quantified dorsal coloration of 328 specimens of *Z. luteus* and *Z. princeps* to explore drivers of intra and interspecific variation with the objective of answering the following questions: Do populations of *Z. luteus* and *Z. princeps* differ in coloration? Do the two species differ in sympatry as compared to allopatry? Can we reliably differentiate between the two species in sympatry based on coloration? And what are the environmental correlates of color differences? By simultaneously exploring patterns within and among species, this work contributes to a greater understanding of how phenotypic variation accumulates and is maintained in nature.

## 2 Materials and Methods

### 2.1 Study Area

**FIGURE 2:**
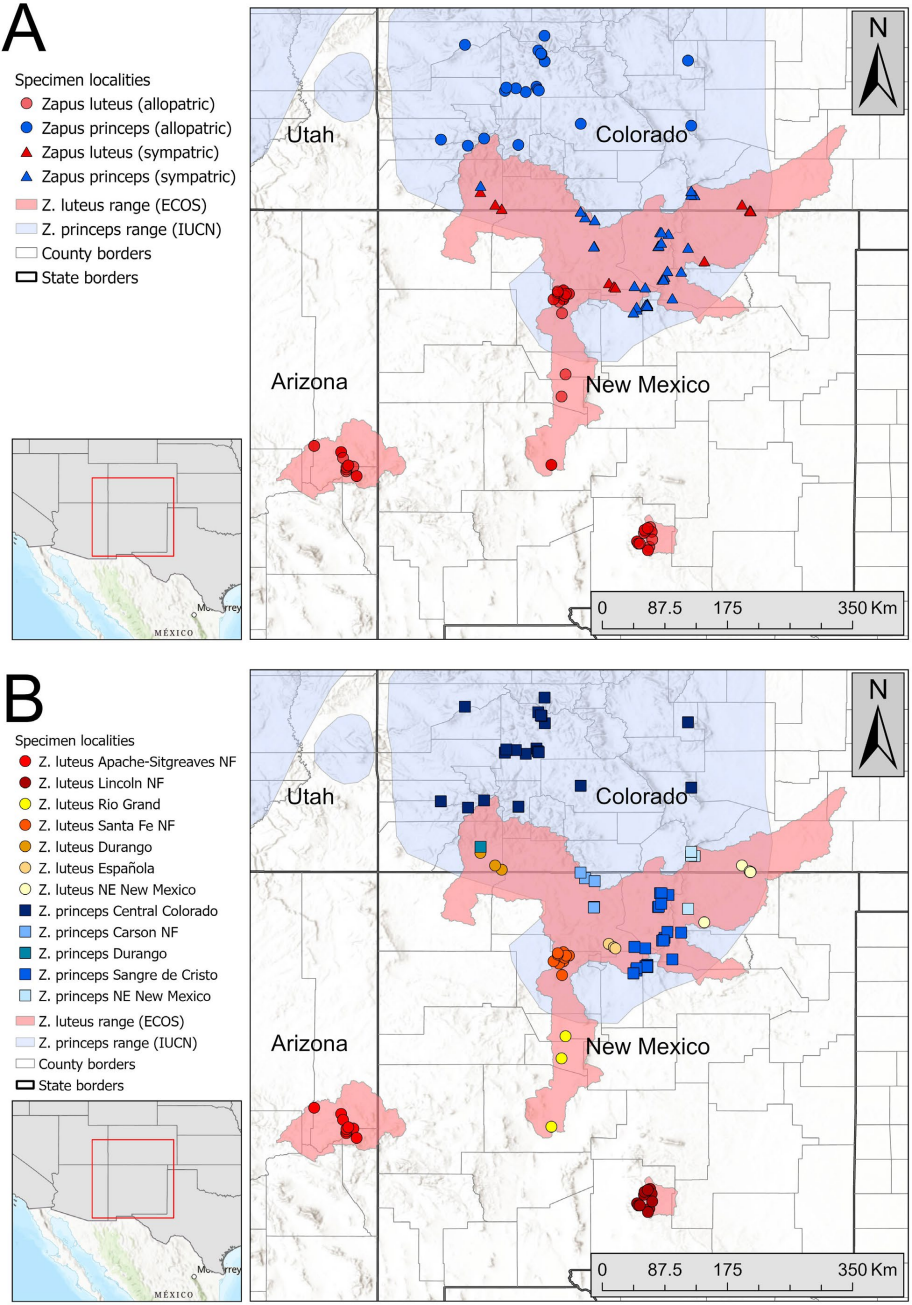
(A) Species ranges and localities of examined specimens of *Zapus luteus* (red; ECOS: https://ecos.fws.gov/ecp/species/7965) and *Z. princeps* (blue; from the International Union for Conservation of Nature [Cassola 2025]). Circles represent allopatric populations and triangles represent sympatric populations. (B) Geographically distinct populations used in the dorsal color analysis of *Z. luteus* (red and yellow) and *Z. princeps* (blue) in Arizona, Colorado, and New Mexico.

Our study area encompassed the known geographic range of *Z. luteus* and a portion of the range of *Z. princeps* in the states of Arizona, Colorado, and New Mexico (Figure 2A; Cassola 2025; Martínez‐Fonseca et al. 2024; USFWS 2020, 2025). We designated 7 population clusters for *Z. luteus* based on known population genetic structure (Figure 2A,B; partially based on USFWS 2020). Populations where *Z. luteus* was the sole *Zapus* species in the area (hereafter allopatric populations) included three isolated high‐elevation areas in the Apache‐Sitgreaves National Forests (ASNF), the Lincoln National Forest (LNF), and the Santa Fe National Forest (SFNF; Jemez Mountains), and one lower elevation population in the Rio Grande (Bernalillo County, Valencia County, and, in Socorro County, Bosque del Apache National Wildlife Refuge). We considered the population on the Santa Fe National Forest as an allopatric area despite being in the sympatric zone of *Z. princeps* based on range polygons (Figure 2). This population has been extensively surveyed and only *Z. luteus* has been captured and confirmed genetically (Sanchez 2021; Martínez‐Fonseca et al. 2025), and there are no historic records of *Z. princeps* in the area. All of these allopatric populations are well defined as separate populations by the U.S. Fish and Wildlife Service (USFWS 2020, 2023).

Populations where *Z. luteus* co‐occur with *Z. princeps* (hereafter sympatric populations) included northeastern New Mexico and southern Colorado in Colfax and Las Animas Counties (Lake Dorothey State Wildlife Area and Sugarite Canyon; hereafter “NE New Mexico”) and in southwestern Colorado in Archuleta and La Plata Counties (hereafter “Durango”). Finally, we included three specimens from Española in Rio Arriba County, New Mexico, which were geographically isolated in a valley from the closest populations in the SFNF.

For *Z. princeps* , we sampled five populations in northern New Mexico and southern Colorado where the species overlapped with *Z. luteus* (Figure 2A,B). When possible, we compared sympatric populations of *Z. princeps* to the closest population of *Z. luteus* . Most of the specimens of *Z. princeps* in the sympatric zone were from the Sangre de Cristo Mountains (separated from the other Lower Rocky Mountains in New Mexico by the Rio Grande Rift) in Santa Fe, San Miguel, Mora, Rio Arriba, and Taos Counties. We separated lower elevation records from Colfax and Las Animas County (hereafter “NE New Mexico”). We also include specimens from the Carson National Forest (lower San Juan Mountains; Rio Arriba County). In southwestern Colorado, we include specimen records in syntopic sites in La Plata County (hereafter “Durango”). Finally, we included allopatric populations of *Z. princeps* from central Colorado, leaving a buffer of at least 80 km north of the closest *Z. luteus* records to avoid any overlap with the sympatric zone. Unlike *Z. luteus* , no distinct populations were defined for *Z. princeps* within our study area, so we used records from central Colorado that approximated the latitudinal and longitudinal expanse of the allopatric *Z. luteus* in the south.

### 2.2 Color Data

**FIGURE 3:**
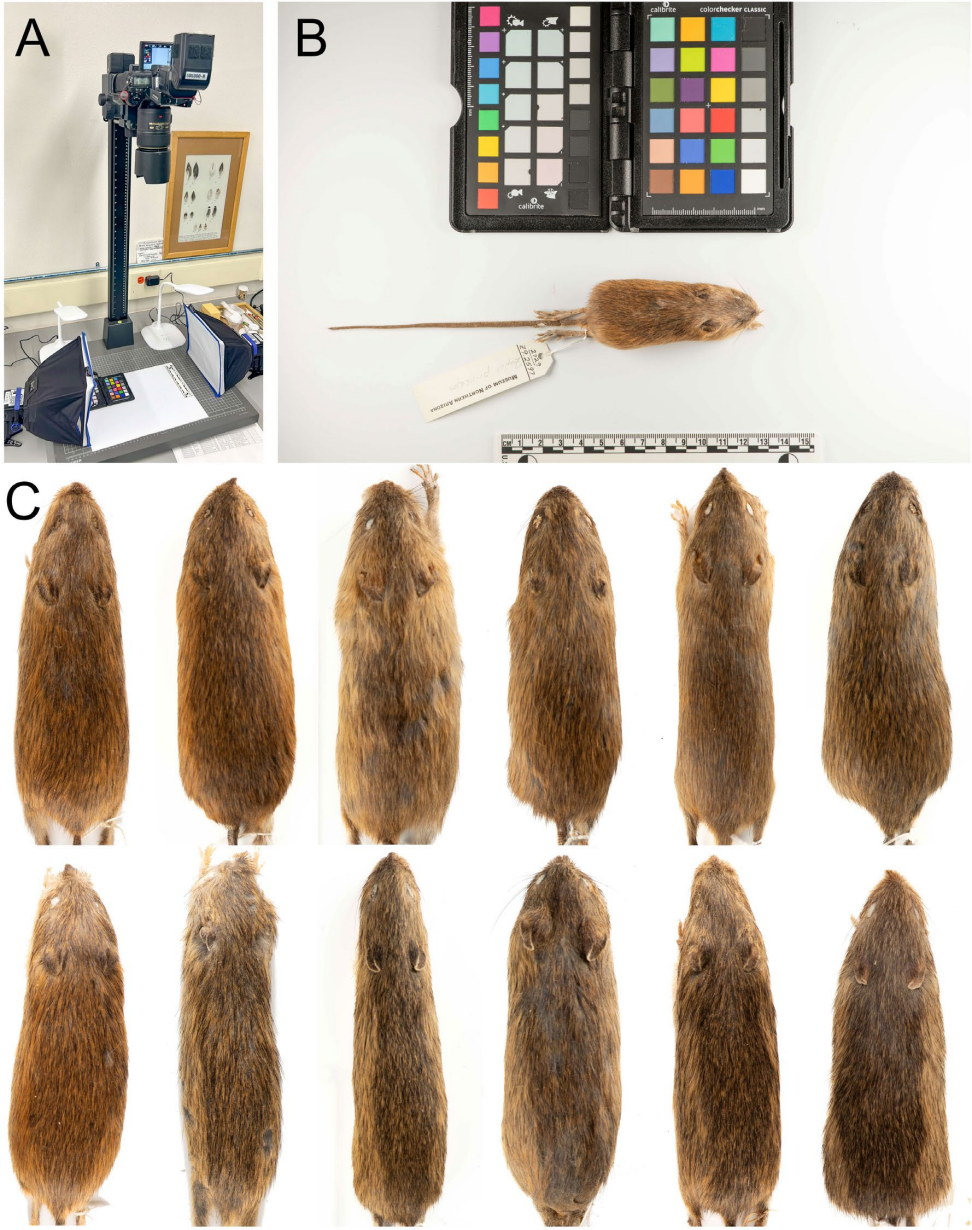
(A) Camera (Nikon D780, 105 mm lens) and lighting (two Nikon SB‐5000) setup used to photograph skins of *Z. luteus* with *Z. princeps* and extract hue, saturation, and value. (B) Examples of an unprocessed specimen photograph from photography setup (MNA Z92597). (C) Color corrected images showing phenotypic variation across *Zapus* spp. in Arizona, Colorado, and New Mexico (Top row from left to right: *Z. luteus* from Navajo Co. [MVZ56822], Otero Co. [MSB37323], Valencia Co. [MSB62103], Sandoval Co. [MVZ56822], Rio Arriba Co. [MSB58370], La Plata Co. [MSB325322]. Bottom row left to right: *Z. luteus* from Las Animas Co. [DMNS8630]; *Z. princeps* from Las Animas Co. [DMNS10875], Taos Co. [MSB35653], Rio Arriba Co. [MSB37670], La Plata Co. [MVZ61304], Teller Co. [UCM19334]).

To gather color data, we photographed skins of both *Z. princeps* and *Z. luteus* available in museums. We did not use any wet specimens. All skins were preserved and stored in climate‐controlled environments at research institutions. While the exact procedure for skin preparation is not recorded with each specimen, preservation methods are relatively standardized within museums and research institutions in the United States (Hall 1962; Williams and Hawks 1987; Dunnum et al. 2017). We photographed the skins using a DSLR camera (Nikon D780, Nikon Inc. Japan) at 1/200 s shutter speed and ISO100 (base), with a 105 mm macro lens and an aperture setting of f11‐13 to eliminate the effect of ambient light. To maintain color accuracy, even illumination was obtained by using two speedlights (Nikon SB‐5000) at a nominal 5500 K color temperature, each with fitted softboxes (Ezybox, Lino Manfrotto + Co. Spa, Italy) to reduce glare and shadows (Figure 3A). Skins were taken from storage directly to the photography setup without manipulation. We photographed all specimens using a white background and included in the framing a ColorChecker Passport Photo 2 (Figure 3B; X‐rite, Grand Rapids, MI) for reference. We photographed the dorsal and ventral surfaces of each specimen in a consistent orientation parallel to the two softboxes to minimize light gradients and shadows. Flash power was also adjusted individually to achieve even illumination (usually between 1/16 and 1/8 power setting). After importing, all photos were corrected using the ColorChecker in Adobe PhotoShop CC (Figure 3C; Adobe Inc., San Jose, CA).

In total we photographed 132 skins of *Z. luteus* and 196 of *Z. princeps* from the mammal collections of the Museum of Southwestern Biology at the University of New Mexico (MSB; *n* = 236), the UC Berkeley Museum of Vertebrate Zoology (MVZ; *n* = 34), the Museum of Northern Arizona (MNA; *n* = 8), the University of Colorado Museum of Natural History (UCM; *n* = 26), and the Denver Museum of Nature and Science (DMNS; *n* = 26). For *Z. luteus* , specimens were assigned to 7 discrete populations: ASNF (*n* = 40), Durango (*n* = 4), Española (*n* = 3), LNF (*n* = 33), NE New Mexico (*n* = 15), Rio Grande (*n* = 15), and SFNF (*n* = 22). For *Z. princeps* , specimens were assigned to the allopatric group in central Colorado (*n* = 50), CNF (*n* = 15), Durango (*n* = 9), Sangre de Cristo Mountains (*n* = 112), and NE New Mexico (*n* = 11). We grouped specimens into populations based on geographic location and not the museum from which the specimen was sourced. We used the associated geographic coordinate data (WGS84) and ArcGIS pro (Esri Inc. Redlands, CA) to locate the collecting site and assign them to the discrete populations previously described. The full dataset of the specimens used in this study and additional specimens of Zapodidae from North America with HSV values, bioclimate data for their locations, and links to the corresponding Arctos and VertNet entries can be found in Appendix S1.

**FIGURE 4:**
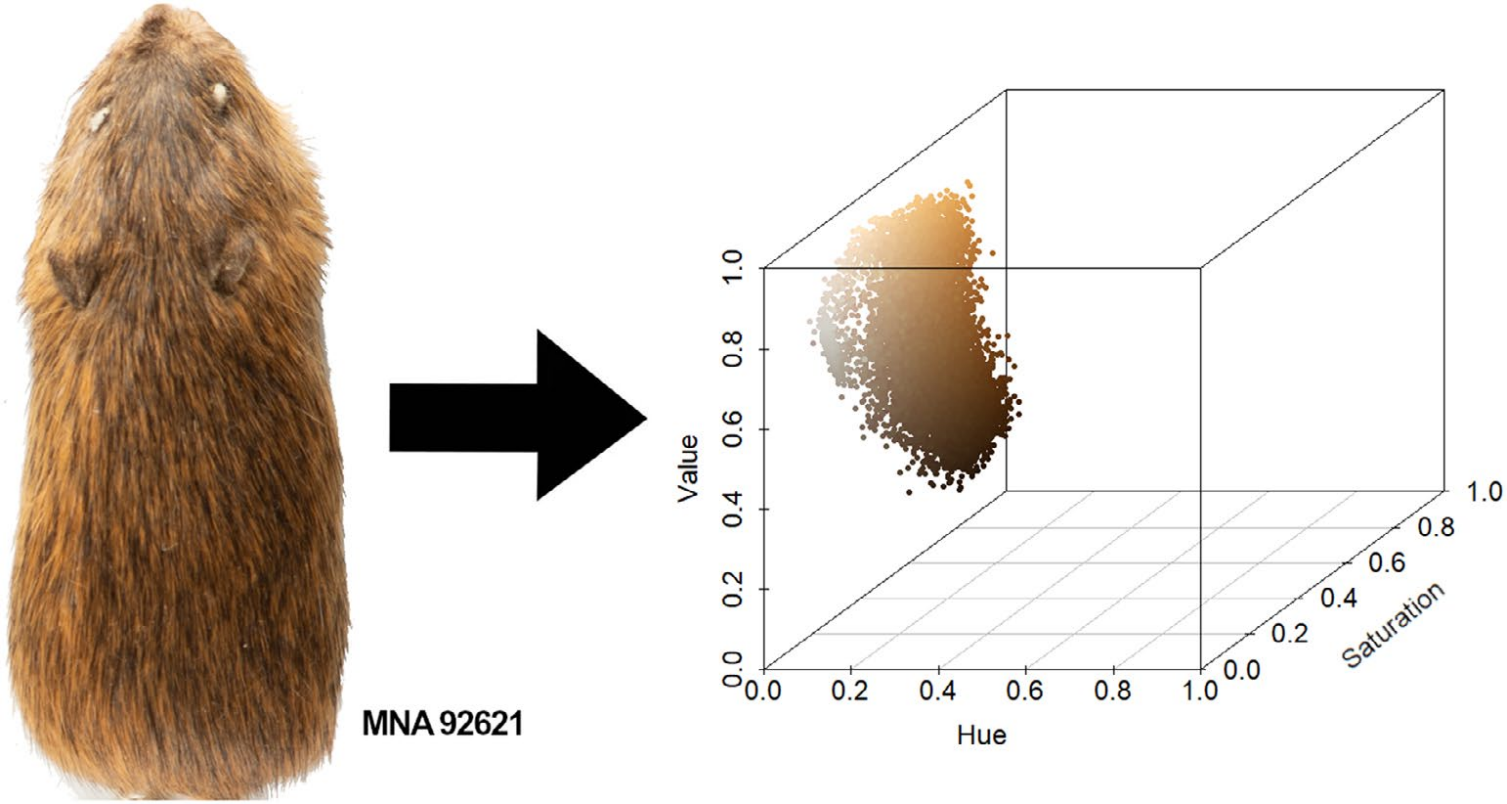
Visual representation of the quantification of dorsal color of a specimen of *Zapus luteus* (MNA 92621; Apache‐Sitgreaves National Forests) as its constituent components of the dominant wavelength, hue (H), the saturation or purity (S), and value (V; a measure of brightness). Each point represents a single pixel from the adjacent photograph and its location in 3‐dimensional color space (Weller and Westneat 2019).

After standardizing photographs of specimens, we imported them into R v. 4.3.1 (R Core Team 2023) and used the function “getHistList” in the package “colordistance” to quantify coloration from specimen photos (Weller and Westneat 2019). We quantified dorsal color using the hue, saturation, value (HSV) approach that separated color into three intuitive and visibly distinct components (Figure 4). Hue (H) represented the dominant wavelength measured in degrees (0–360); saturation (S) or purity measured the purity on intensity of the color scale 0–1 from least intense/saturated to fully saturated; and value (V), a measure of brightness, on a scale from black (0) to white (1). HSV measures of color more closely approximate vertebrate color vision than other color spaces such as red, green, blue (Endler and Mielke 2005; Endler 2012). Value is of particular interest because it is used in studies of crypsis and predator detection (Gillis 1989). We focused on dorsal photographs because the dorsum is exposed to predators and competitors. We also found that the condition of the ventral surface of these specimens was not always uniform (e.g., visible stitches on the specimen skin, missing areas, hair loss, fill material exposed).

### 2.3 Statistical Analyses

1. Do populations of *Z. luteus* and *Z. princeps* differ in coloration?

We used analysis of variance (ANOVA) to assess intraspecific differences in color using the “aov” function in the package “stats” (R Core Team 2023). For each species, we grouped individuals into populations as stated above and shown in Figure 2B. We ran this analysis separately for the two species with distinct populations tested separately. Prior to ANOVA we checked the assumption of normality using histograms and the assumption of equal variances by computing per‐population standard deviations. When ANOVAs were significant, we performed post hoc comparisons using Tukey's honest significance differences (HSD) via the “TukeyHSD” function again in the package “stats.” To estimate effect sizes, we calculated eta‐squared (*η* ^2^) values which are used for ANOVA and can be interpreted like *R* ^2^ values. We used the “eta_squared” function in the package “rstatix” (Kassambara 2023).

For *Z. luteus* , we grouped individuals by population using the Species Status Assessment (SSA) main subpopulation delimitations that rely on the 8th Hydrologic Unit Code (HUC8; USFWS 2020, 2023). For *Z. princeps* , we defined populations using four well‐defined geographic features which included specimens from higher elevations in the CNF and Sangre de Cristo Mountains. We also included a separate population in Durango along the Florida River (La Plata County, Colorado) and a lower elevation population in NE New Mexico (including a few across the state border in southern Colorado in Las Animas and Colfax Counties).

2 Do species differ in coloration in sympatry?

To look for evidence of character displacement, we grouped species based on geographic overlap (sympatric or allopatric) and tested for differences in brightness using ANOVA. Prior to ANOVA we checked the assumption of normality using histograms and the assumption of equal variances by computing per‐population standard deviations. When ANOVAs were significant, we performed post hoc comparisons, and to estimate effect sizes, we calculated *η* ^2^. If character displacement has occurred, we would expect the magnitude of color difference between the two species in sympatry to be greater than the difference in allopatry. Alternatively, if habitat filtering occurred, we would expect the sympatric populations to be more similar than allopatric populations.

3 Can we statistically differentiate between the two species based on coloration?

We used Linear Discriminant Analysis (LDA) executed in the “MASS” package (Venables and Ripley 2002) to assess whether HSV color data was a reliable predictor of species assignment where the two species occurred in sympatry. We used all three color axes (hue, saturation, and value) in this analysis. We computed the accuracy of the LDA‐predicted species assignments compared to the true species assignments. Because our sample sizes in sympatry were uneven ( *Z. luteus* = 22, *Z. princeps* = 146), we also conducted a random sampling of 22 *Z. princeps* individuals and re‐ran the LDA prediction with equal sample sizes for both species. We repeated this process 1000 times and stored the accuracy to assess how reliably we could predict species in sympatry based on their coloration.

4 What are the environmental correlates of color differences in *Z. luteus* and *Z. princeps* ?

We constructed generalized linear models (GLM) to assess factors influencing dorsal brightness across populations for both *Z. luteus* and *Z. princeps* . We extracted bioclimatic variables (hereafter, “bioclim”) at 30 arc sec resolution (~1 km) from the CHELSA global climate dataset (Karger et al. 2017; Brun et al. 2022) at all specimen locations using the “extract” function in the package “raster” (Hijmans and van Etten 2012; Karger et al. 2017; Brun et al. 2022). To reduce the dimensionality of the 19 bioclim variables, we conducted a principal components analysis (PCA; McGarigal et al. 2000) of the climate values at all points. Temperature seasonality (Bio4) loaded highly onto PC1 (loading: 0.98) and annual precipitation (Bio12) loaded highly onto PC2 (loading: −0.85). The first two PC axes together accounted for 96.5% of the variance in climate across specimen locations. Therefore, we included Bio4 and Bio12 in our models as measures of climate variation. We used the actual bioclimatic variables rather than composite PC axes in our models to aid in interpretation. Models contained the following additional covariates: elevation, latitude, sex, and collection year. We additionally included population ID as a random factor in our models. We examined only adult specimens, so neither age nor body size was included.

We scaled our response variable, brightness, and all quantitative predictor variables (elevation, latitude, Bio4, Bio12, and collection year) using a z‐transformation which rescaled data to a mean of 0 and a standard deviation of 1 so that we could directly compare the relative importance of each predictor variable on dorsal brightness (R Core Team 2023). For model selection, we used the “MuMIn” package, which generated a model selection table with subsets of fixed effects terms in the global model that were ranked and evaluated using Akaike's Information Criterion adjusted for small sample size (AICc; Bartón 2022). We used model averaging to average parameter estimates across all models with ΔAICc ≤ 4 (Anderson 2008) and included full and null models for reference. We also included the number of models containing each variable from our subset models as a measure of variable strength (Anderson 2008).

## 3 Results

1. Do populations of *Z. luteus* and *Z. princeps* differ in coloration?

**FIGURE 5:**
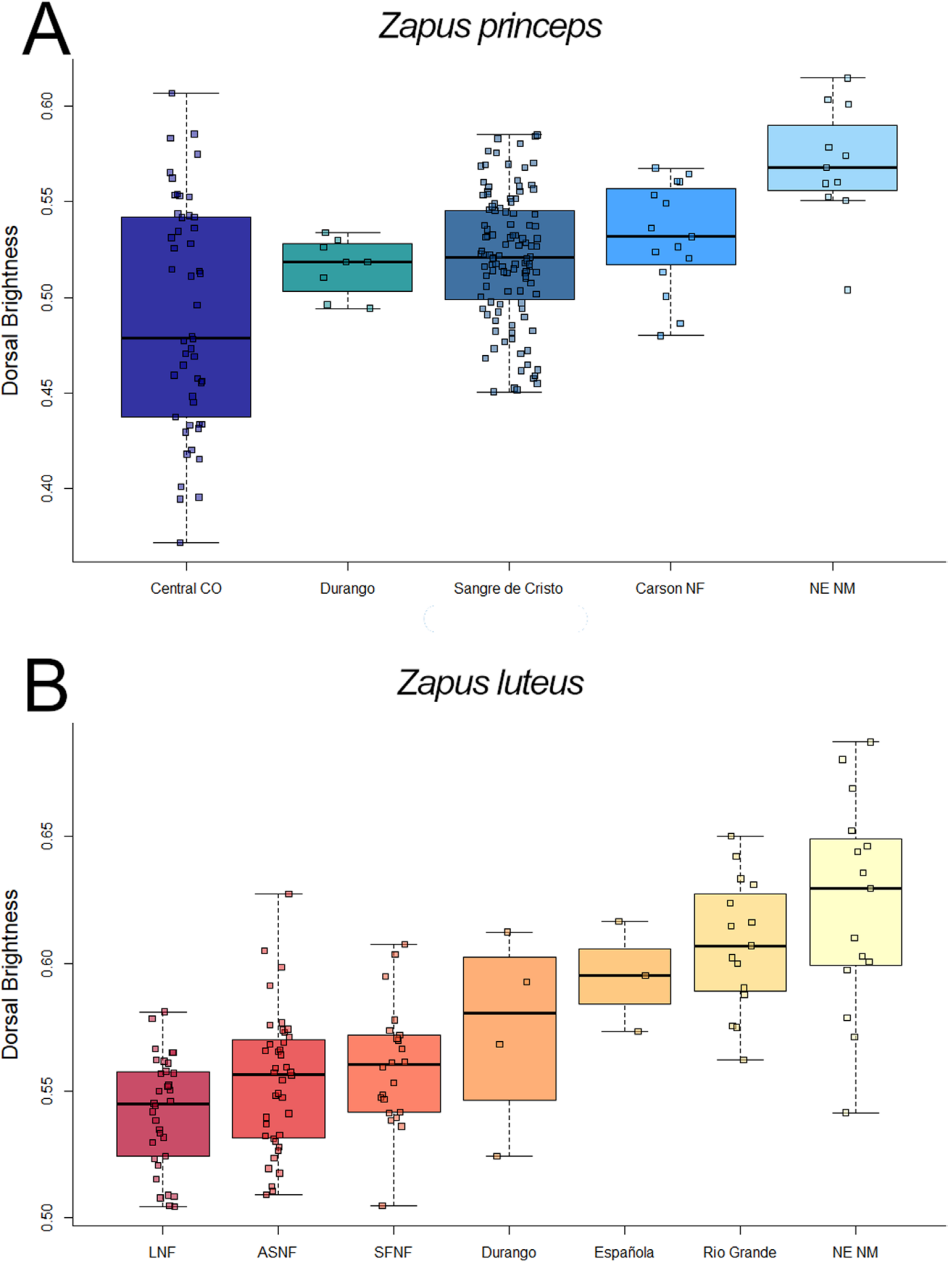
Variation in dorsal pelage brightness across populations for two species of jumping mouse (*Zapus* spp.) in Arizona (Apaches‐Sitgreaves National Forests [ASNF]), Colorado (Durango, Central Colorado [Central CO]), and New Mexico (Carson National Forest [Carson NF]; Española, Lincoln National Forest [LNF]; Sangre de Cristo, Santa Fe Nacional Forest [SFNF], Rio Grande, northeastern New Mexico [NE NM; includes nearby specimens in Colorado]). (A) *Z. princeps* and (B) *Z. luteus*.

Populations of *Z. princeps* differed significantly in brightness (Figure 5A; ANOVA, *F* = 10.68, *p* = 0.0001, *η* ^2^ = 0.183). In our post hoc comparisons (Tukey's HSD), populations in the CNF, Sangre de Cristo Mountains, and NE New Mexico were significantly brighter than the population in central Colorado (*p* = 0.006). We also found that the population in NE New Mexico was significantly brighter than the populations in Durango and Sangre de Cristo Mountains (*p* = 0.03).

Populations of *Z. luteus* also differed in brightness (Figure 5B; *F* = 22.82, *p* = 0.0001, *η* ^2^ = 0.536). The NE New Mexico population was brighter than populations on the ASNF (*p* = 0), in Durango (*p* = 0.033), the LNF (*p* = 0), and the SFNF (*p* = 0). The Rio Grande population was similarly brighter than populations on the ASNF (*p* = 0), the LNF (*p* = 0), and the SFNF (*p* < 0.0001). Finally, the population in Espanola was brighter than that in the LNF (*p* = 0.027).

2 Do species differ in coloration in sympatry?

**FIGURE 6:**
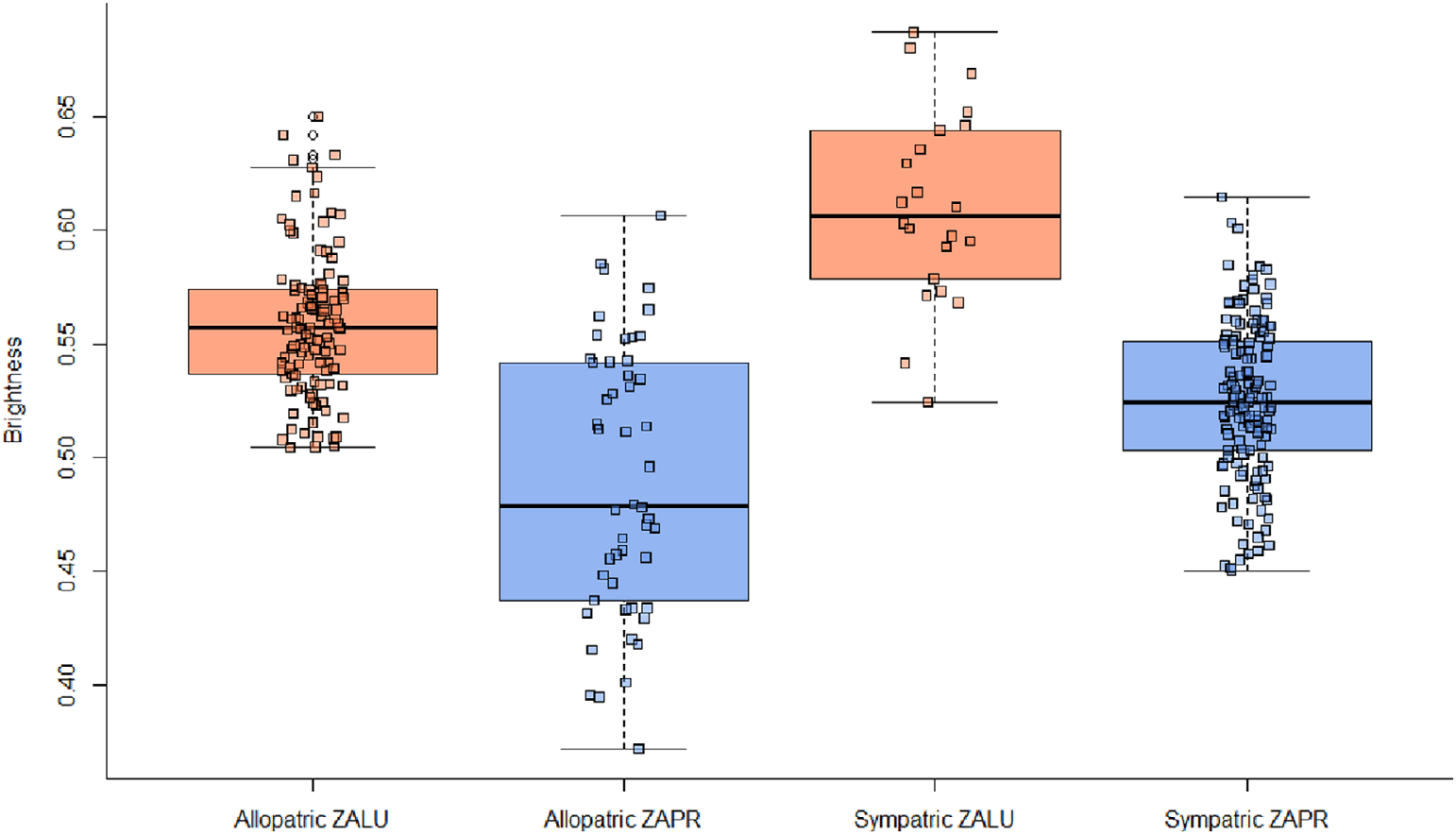
Differences between dorsal pelage brightness in sympatric and allopatric individuals of *Zapus princeps* (ZAPR) and *Z. luteus* (ZALU) in Arizona, Colorado, and New Mexico.

Allopatric and sympatric populations of *Z. luteus* and *Z. princeps* differed significantly in brightness (ANOVA, *F* = 65.93, *p* < 0.0001, *η* ^2^ = 0.379). Populations of both species were brighter in areas of sympatry than their respective allopatric populations; for *Z. luteus*, the difference in brightness was 0.051, and for *Z. princeps* , the difference was 0.034 (Figure 6). Post hoc analyses revealed that populations of *Z. luteus* and *Z. princeps* were significantly different in areas of sympatry (*p* = 0). Additionally, the magnitude of difference in mean brightness between sympatric *Z. luteus* and *Z. princeps* was greater (0.085) than the difference in allopatry (0.068).

3 Can we statistically differentiate between the two species based on coloration?

Species assignment based on HSV color had an overall accuracy of 94.64% (LDA). The coefficients of the linear discriminants were as follows: hue = 57.09, saturation = −10.63, brightness = −20.53. *Zapus luteus* was correctly predicted 15 out of 22 times, while *Z. princeps* was predicted correctly 144 out of 146. Our resampling procedure produced a median accuracy of 95.45% with a standard deviation of 0.0239, or 2.39%.

4 What are the environmental correlates of color differences in *Z. luteus* and *Z. princeps* ?

For *Z. princeps* , the top model (GLM, *w*_*i*_ = 0.65; Table 1) included all covariates except sex (Table 2). Temperature seasonality (Bio4), annual precipitation (Bio12), elevation, and latitude were included in all models with ΔAICc ≤ 4. Bio4 was positively associated with brightness (*β =* 0.3180; SE = 0.0988; *p* = 0.001), as was elevation (*β =* 0.3488; SE = 0.1084; *p* = 0.001). Bio12 was negatively associated with individual brightness (*β =* −0.3906; SE = 0.0998) as was latitude (*β = −*0.3475; SE = 0.0856; *p* < 0.001; brighter at lower latitudes). Animals with brighter pelage were found in areas with greater temperature seasonality and at higher elevations but with lower annual precipitation and latitude.

**TABLE 1:** Top models for dorsal pelage brightness from photographs of museum specimens of jumping mice ( *Z. princeps* and *Z. luteus* ) in Arizona, Colorado, and New Mexico.

Model	df	logLik	AICc	ΔAICc	*w*_*i*_
*Zapus princeps*					
Brightness ~ Bio4 + Bio12 + collection year + elevation + latitude	7	−258.72	532.05	0.00	0.65
Brightness ~ Bio4 + Bio12 + collection year + elevation + latitude + sex (full model)	8	−258.64	534.06	2.00	0.24
Brightness ~ Bio4 + Bio12 + elevation + latitude	6	−261.54	535.52	3.47	0.11
Brightness ~. (null model)	2	−273.35	550.80	18.72	0.00
*Zapus luteus*					
Brightness ~ Bio4 + elevation + latitude	5	−157.15	324.77	0.00	0.24
Brightness ~ Bio4 + collection year + elevation + latitude	6	−156.70	326.08	1.31	0.12
Brightness ~ Bio4 + elevation + latitude + sex	6	−156.94	326.54	1.77	0.10
Brightness ~ Bio4 + Bio12 + elevation + latitude	6	−157.14	326.94	2.17	0.08
Brightness ~ Bio12 + elevation + latitude	5	−158.27	327.02	2.25	0.08
Brightness ~ elevation + latitude	4	−159.40	327.11	2.33	0.07
Brightness ~ collection year + elevation + latitude	5	−158.37	327.21	2.43	0.07
Brightness ~ Bio12 + collection year + elevation + latitude	6	−157.60	327.87	3.09	0.05
Brightness ~ Bio4 + collection year + elevation + latitude + sex	7	−156.59	328.08	3.31	0.05
Brightness ~ Bio4 + Bio12 + collection year + elevation + latitude	7	−156.69	328.28	3.50	0.04
Brightness ~ elevation + latitude + sex	5	−159.09	328.66	3.88	0.03
Brightness ~ Bio12 + elevation + latitude + sex	6	−158.03	328.74	3.97	0.03
Brightness ~ Bio4 + Bio12 + elevation + latitude + sex	7	−156.92	328.74	3.97	0.03
Brightness ~ Bio4 + Bio12 + collection year + elevation + latitude + sex (full model)	8	−156.57	330.30	5.54	0.01
Brightness ~. (null model)	2	−186.79	377.7	52.91	0.00

*Note:* Models were ranked based on Akaike's Information Criterion adjusted for small sample size (ΔAICc ≤ 4.0), *w*_*i*_ = AICc weight, and logLik = log likelihood. Global (full) and null (.) models were included for comparison. Models included population as a random effect. Temperature seasonality = Bio4; annual precipitation = Bio12.

**TABLE 2:** Variables in models (ΔAICc ≤ 4) of dorsal pelage brightness of *Zapus princeps* and *Z. luteus* in Arizona, Colorado, and New Mexico.

Model	*β*	SE	*w*_*ij*_	Containing models
*Zapus princeps*				
Temperature seasonality (Bio4)	0.3180	0.0988	1.00	3/3
Annual precipitation (Bio12)	−0.3906	0.0998	1.00	3/3
Elevation	0.3488	0.1084	1.00	3/3
Latitude	−0.3475	0.0856	1.00	3/3
Collection year	−0.1836	0.1062	0.89	2/3
Sex	−0.0134	0.0713	0.24	1/3
*Zapus luteus*				
Elevation	−0.4876	0.1238	1.00	13/13
Latitude	0.6242	0.1919	1.00	13/13
Temperature seasonality (Bio4)	−0.2496	0.2496	0.66	7/13
Collection year	−0.0277	0.0612	0.33	5/13
Annual precipitation (Bio12)	0.0224	0.1102	0.32	6/13
Sex	0.0222	0.0816	0.24	5/13

*Note:* This table includes full model‐averaged coefficients (*β*), unconditional standard errors (SE), and cumulative weights (*w*_*ij*_; full average).

For *Z. luteus* , the top model (GLM, *w*_*i*_ = 0.24; Table 1) included Bio4, elevation and latitude. Only elevation and latitude are included in all models with ΔAICc ≤ 4. Brightness values of pelage for *Z. luteus* were negatively correlated with elevation (*β =* −0.4876; SE = 0.1238; *p* < 0.001) and positively correlated with latitude (*β =* 0.6442; SE = 0.1919; *p* = 0.001; Table 2). Temperature seasonality, annual precipitation, year of collection, and sex were not significant (*p* > 0.3). For this species, animals with brighter pelage were found at lower elevations and higher latitude.

## 4 Discussion

1. Do populations of *Z. luteus* and *Z. princeps* differ in coloration?

We found variation in dorsal brightness within and between populations of *Z. luteus* and *Z. princeps* in the southwestern United States. For both species, the populations with the brightest coloration occurred in northeastern New Mexico closer to the Great Plains where geology, plant structure, and species composition might differ the most from the other parts of their range (Hall and Penner 2013) (Figure 5). Populations of *Z. luteus* generally differed between some low (Rio Grande, NENM) and high elevation (ASNF, LNF, and SFNF) sites, supporting previous evidence of local adaptation between populations (Malaney et al. 2012, 2017; Sanchez 2021). Likewise, *Z. princeps* showed statistically significant differences in northern New Mexico and adjacent allopatric populations in central Colorado. With the exception of the populations of *Z. luteus* along the Rio Grande, all other populations of *Z. luteus* and *Z. princeps* that we studied occurred at higher elevations and more mountainous terrain with more open and short vegetation.

In the case of *Z. luteus* , these differences in color between populations are consistent with evidence of genetic differentiation, especially among isolated populations of eastern Arizona and central New Mexico (Malaney et al. 2012, 2017; Sanchez 2021). This phenotypic differentiation is also the first to our knowledge to be documented across populations of *Z. luteus* . Evidence like this might become important for conservation of distinct populations in the future, as it is for the closely‐related *Z. preblei* (King et al. 2006).

2 Do species differ in coloration in sympatry?

Both species of *Zapus* increased in dorsal brightness where they occur in sympatry, which could indicate some degree of habitat filtering (Figure 6). However, the difference in brightness between the two species was also larger when in sympatry. This relative divergence in brightness at the sympatric zones of *Z. luteus* and *Z. princeps* in southern Colorado and northern New Mexico could suggest that character displacement has occurred. This divergence in dorsal brightness could be an indication of differential predation pressure, differences in preferred microhabitat, behavior, or intraspecific recognition. Both species of *Zapus* construct bolus day nests and these could vary depending on the available materials at the site which, in turn, could selectively affect dorsal color by natural selection processes.

Brightness differences between the two species in sympatry are above the average threshold of perceptibility to humans (Vadivel et al. 2005). However, human perception is difficult to generalize and other factors of the observer (e.g., age, visual acuity, color vision deficiencies, memory) as well as environmental conditions (e.g., type and quality of available light) could affect the ability to discern these differences reliably (Emery and Webster 2019).

3 Can we statistically differentiate between the two species based on coloration?

Statistical models predicted species assignments with 95% accuracy using HSV color data, however these differences do not necessarily reflect the difficulty of field identification. In the area of sympatry, *Z. luteus* revealed a greater difference to other populations of its own species than did *Z. princeps* . This could be the result of stronger geographic isolation in the valleys (lower elevation) for *Z. luteus* relative to *Z. princeps* , which is overall more continuously distributed across high elevation mountain ranges. Despite the subtle differences in color that might make identification by humans challenging, these brightness differences could be significant to visual predators (Endler and Mielke 2005; Endler 2012). Examining discrete features (e.g., stripes, color fringes, ear tufts) might help reliably visually discriminate between the two species during field surveys.

4 What are the environmental correlates of color differences in *Z. luteus* and *Z. princeps* ?

Relationships between environmental variables and brightness differed between the two species and with the exception of collection year, all responses to elevation, latitude, sex, temperature seasonality, and annual precipitation were opposite. We found that elevation had an inverse relationship with brightness in *Z. luteus* , a pattern consistent with Bogert's rule (thermal melanism) and observed in many other taxa including mammals, birds, and arthropods (Delhey et al. 2019; Cerezer et al. 2024). However, latitudinal effects in *Z. luteus* suggest a pattern more in accordance with Gloger's rule, where organisms at higher latitudes and thus colder climates were brighter (Delhey 2019; Delhey et al. 2019; Tian and Benton 2020). We did not observe this pattern in *Z. princeps*; however, this species has a larger geographic distribution than *Z. luteus* and we have only studied specimens from the southern portion of its range. Additionally, this species is more of a habitat generalist than *Z. luteus* . *Zapus princeps* commonly occurs in mesic areas but can be found away from riparian areas if soil moisture levels are high (Blake Hart et al. 2004). Environmental pressures could be substantially different for *Z. princeps* compared to the riparian‐obligate, *Z. luteus* , an animal that remains within a mean of 12 m of a stream or river system (C. Chambers, unpublished data).

Temperature seasonality (Bio4) was positively and strongly correlated with brightness in *Z. princeps* . High elevation sites experience greater seasonality including snow and the loss of vegetation in winter, with vegetation cover being overall lower due to shorter growing seasons and brighter understory. These factors could favor increased brightness in *Z. princeps* where it occurs at high elevation. Annual precipitation (Bio12) was also strongly and negatively correlated with brightness in *Z. princeps*. Gloger's rule posits that animals should be more pigmented in warmer and wetter environments, and we find support for that pattern in *Z. princeps*. Finally, sex and collection year were contained in the fewest models across both species, suggesting that environmental variables (e.g., resource availability, predation risk, and habitat characteristics) are better predictors of color in these species than sexual dimorphism and year of specimen preparation. Sexual differences in body size have been reported for *Z. luteus* (Frey 2008) but we did not detect significant differences in coloration associated with sex.

### 4.1 Opportunities for Future Study

Our analysis could be improved with the addition of more specimens, especially from the areas of sympatry and especially syntopy that are currently poorly represented by specimen material. These populations include those of *Z. luteus* and *Z. princeps* in La Plata and Rio Arriba Counties (e.g., Durango, Española). The endangered status of *Z. luteus* poses a challenge in collecting specimens from areas of sympatry with *Z. princeps* . However, with advances in imaging technology, we hope that additional phenotypic data including coloration and morphology could be obtained from these areas to provide tools for discerning species and populations. Live animals could be photographed under standardized lighting conditions during surveys and contribute phenotypic data for these and other overlapping *Zapus* species (*Z. preblei* and *Z. princeps* ). Increased sample sizes within and among populations might also help detect subtle differences in phenotypic characters. Furthermore, non‐invasive DNA sample information could be coupled with photographs to further improve and train photograph‐only species identification in sympatric zones.

Additional color and natural history information of sympatric *Zapus* species and their predators could open new areas of study. Simulating different predator vision (e.g., coyote, hawk, snake) in different environments and light spectrums (e.g., ultraviolet, near infrared) could provide insights into the role that crypsis plays in this system. Likewise, additional information on microhabitat use and background or substrate color could clarify whether differences in color among populations or species are due to the differences in the surrounding geology and plant communities. Studies examining behavior and competition between the two species will improve our understanding of the role of color in interspecific recognition or niche partitioning.

Accurate discrimination of the federally endangered *Z. luteus* is important for conservation and management of the species. Considering the individual color variation, and only partial divergent coloration of both *Zapus* species in their sympatric zone, we recommend that more than one criterion be used to determine species with live animals in capture‐release surveys. We should also aim to develop field keys that make use of discrete characteristics that could help species identification based on individuals from populations in sympatry. Use of DNA confirmation from non‐invasive methods like fecal collection or buccal swabs might be necessary to provide reliable identification of new populations (Sanchez 2021; Lyman et al. 2022). Behavioral studies (e.g., nest construction, prey–predator interactions, activity periods, multi‐modal locomotion) could also shed light on the ecological implications of dorsal coloration among *Zapus* species. Furthermore, this divergence in the phenotype of jumping mice populations highlights the genetic diversity and the need to conserve these in the face of climate change and other stochastic events that will require fast adaptation to the changing environment.

## 5 Conclusions

We found that dorsal brightness in two co‐occurring jumping mice species varies within and between populations, shaped by both environmental factors and interspecific interactions. *Zapus luteus* showed brightness patterns consistent with thermal melanism, while *Z. princeps* exhibited associations with precipitation and temperature seasonality. In sympatry, both species were brighter and showed greater interspecific divergence than in allopatry, suggesting a role for both shared environmental pressures and character displacement. Although statistical models classified species with high accuracy, brightness differences are subtle and not always reliable for field identification. Future research should expand sampling in poorly represented sympatric zones, integrate standardized photography of live animals, and simulate predator vision to assess detectability across environments. Overall, our findings demonstrate how both abiotic and biotic pressures drive divergent phenotypic evolution in closely related species.

## Author Contributions

**José Gabriel Martínez‐Fonseca:** conceptualization (equal), data curation (equal), formal analysis (supporting), visualization (equal), writing – original draft (equal), writing – review and editing (equal). **Erin P. Westeen:** conceptualization (equal), data curation (equal), formal analysis (lead), writing – original draft (equal), writing – review and editing (equal). **Jennifer L. Zahratka:** conceptualization (equal), writing – review and editing (equal). **Carol L. Chambers:** conceptualization (equal), funding acquisition (equal), supervision (lead), writing – review and editing (supporting).

## Conflicts of Interest

The authors declare no conflicts of interest.

## Supporting information


**Appendix S1:** Specimens of *Zapus luteus* and *Z. princeps* photographed and extracted color composition data. Also included, bioclimatic data for each of the specimen's localities in Arizona, Colorado, and New Mexico.

## Data Availability

All data used in this manuscript is available in the main body of the manuscript or in Appendix S1.
