# Supplementary material for: Environmental Factors and Co‐Occurrence Patterns Influence Dorsal Brightness in Two Jumping Mice Species in the Western United States
Source: Ecol Evol. 2026 Feb 22;16(2):e73119. doi: 10.1002/ece3.73119 (PMC12928019; doi:10.1002/ece3.73119)
Supplement: Supplementary file 1 — Appendix S1: Specimens of Zapus luteus and Z. princeps photographed and extracted color composition data. Also included, bioclimatic data for each of the specimen's localities in Arizona, Colorado, and New Mexico. [file ECE3-16-e73119-s001.docx]

**Appendix 1.** Specimens of *Zapus luteus* and *Z. princeps* photographed and extracted color composition data. Also included, bioclimatic data for each of the specimen’s localities in Arizona, Colorado, and New Mexico.

| Species | Population Name | GUID | Sex | Collection. year | Lat | Long | Elevation | H | S | V | Bio1 | Bio2 | Bio3 | Bio4 | Bio5 | Bio6 | Bio7 | Bio8 | Bio9 | Bio10 | Bio11 | Bio12 | Bio13 | Bio14 | Bio15 | Bio16 | Bio17 | Bio18 | Bio19 |
| --- | --- | --- | --- | --- | --- | --- | --- | --- | --- | --- | --- | --- | --- | --- | --- | --- | --- | --- | --- | --- | --- | --- | --- | --- | --- | --- | --- | --- | --- |
| *Z. luteus* | ZALU.LNF | MSB:Mamm:61691 | F | 1988 | 32.7278 | -105.651 | 2584 | 0.067053 | 0.486176 | 0.504893 | 89 | 120 | 372 | 7123 | 251 | -71 | 323 | 178 | 75 | 183 | -11 | 518 | 113 | 13 | 80 | 337 | 47 | 272 | 69 |
| *Z. luteus* | ZALU.LNF | MSB:Mamm:61692 | M | 1988 | 32.7278 | -105.651 | 2584 | 0.069445 | 0.45965 | 0.54499 | 89 | 120 | 372 | 7123 | 251 | -71 | 323 | 178 | 75 | 183 | -11 | 518 | 113 | 13 | 80 | 337 | 47 | 272 | 69 |
| *Z. luteus* | ZALU.LNF | MSB:Mamm:61693 | M | 1988 | 32.7278 | -105.651 | 2584 | 0.070532 | 0.52364 | 0.508562 | 89 | 120 | 372 | 7123 | 251 | -71 | 323 | 178 | 75 | 183 | -11 | 518 | 113 | 13 | 80 | 337 | 47 | 272 | 69 |
| *Z. luteus* | ZALU.LNF | MSB:Mamm:61689 | F | 1988 | 32.7721 | -105.634 | 2476 | 0.069898 | 0.476442 | 0.561663 | 95 | 120 | 371 | 7162 | 259 | -65 | 324 | 185 | 82 | 190 | -5 | 441 | 99 | 11 | 81 | 293 | 41 | 234 | 63 |
| *Z. luteus* | ZALU.LNF | MSB:Mamm:61712 | F | 1988 | 32.8112 | -105.755 | 2807 | 0.072808 | 0.479273 | 0.541861 | 76 | 120 | 375 | 7057 | 237 | -83 | 320 | 165 | 104 | 169 | -23 | 520 | 111 | 13 | 75 | 333 | 49 | 260 | 88 |
| *Z. luteus* | ZALU.LNF | MSB:Mamm:61690 | M | 1988 | 32.8112 | -105.772 | 2757 | 0.07054 | 0.507342 | 0.549906 | 79 | 120 | 374 | 7075 | 240 | -80 | 320 | 168 | 108 | 172 | -20 | 488 | 97 | 14 | 66 | 290 | 52 | 229 | 88 |
| *Z. luteus* | ZALU.LNF | MSB:Mamm:61678 | M | 1988 | 32.8398 | -105.79 | 2713 | 0.071281 | 0.499099 | 0.557005 | 82 | 120 | 374 | 7094 | 243 | -77 | 321 | 171 | 111 | 175 | -18 | 561 | 113 | 16 | 67 | 337 | 60 | 264 | 99 |
| *Z. luteus* | ZALU.LNF | MSB:Mamm:61679 | M | 1988 | 32.8398 | -105.79 | 2713 | 0.068317 | 0.464382 | 0.544156 | 82 | 120 | 374 | 7094 | 243 | -77 | 321 | 171 | 111 | 175 | -18 | 561 | 113 | 16 | 67 | 337 | 60 | 264 | 99 |
| *Z. luteus* | ZALU.LNF | MSB:Mamm:61680 | M | 1988 | 32.8398 | -105.79 | 2713 | 0.071519 | 0.51793 | 0.560858 | 82 | 120 | 374 | 7094 | 243 | -77 | 321 | 171 | 111 | 175 | -18 | 561 | 113 | 16 | 67 | 337 | 60 | 264 | 99 |
| *Z. luteus* | ZALU.LNF | MSB:Mamm:61687 | F | 1988 | 32.8398 | -105.79 | 2713 | 0.069552 | 0.456256 | 0.546002 | 82 | 120 | 374 | 7094 | 243 | -77 | 321 | 171 | 111 | 175 | -18 | 561 | 113 | 16 | 67 | 337 | 60 | 264 | 99 |
| *Z. luteus* | ZALU.LNF | MSB:Mamm:61696 | M | 1988 | 32.8606 | -105.6 | 2264 | 0.068749 | 0.476333 | 0.515386 | 108 | 120 | 368 | 7245 | 273 | -54 | 327 | 198 | 95 | 203 | 6 | 456 | 97 | 12 | 75 | 289 | 46 | 232 | 69 |
| *Z. luteus* | ZALU.LNF | MSB:Mamm:61684 | F | 1988 | 32.9469 | -105.703 | 2632 | 0.069552 | 0.524459 | 0.524316 | 85 | 120 | 373 | 7126 | 248 | -74 | 322 | 175 | 115 | 180 | -14 | 550 | 121 | 14 | 77 | 361 | 52 | 280 | 93 |
| *Z. luteus* | ZALU.LNF | MSB:Mamm:37323 | F | 1978 | 32.95754 | -105.687 | 2587 | 0.071094 | 0.529642 | 0.550207 | 88 | 120 | 373 | 7140 | 251 | -71 | 322 | 178 | 118 | 182 | -12 | 502 | 110 | 13 | 77 | 329 | 49 | 253 | 84 |
| *Z. luteus* | ZALU.LNF | MSB:Mamm:37324 | F | 1978 | 32.95754 | -105.687 | 2587 | 0.069806 | 0.413873 | 0.523162 | 88 | 120 | 373 | 7140 | 251 | -71 | 322 | 178 | 118 | 182 | -12 | 502 | 110 | 13 | 77 | 329 | 49 | 253 | 84 |
| *Z. luteus* | ZALU.LNF | MSB:Mamm:37325 | F | 1978 | 32.95754 | -105.687 | 2587 | 0.068005 | 0.468956 | 0.529686 | 88 | 120 | 373 | 7140 | 251 | -71 | 322 | 178 | 118 | 182 | -12 | 502 | 110 | 13 | 77 | 329 | 49 | 253 | 84 |
| *Z. luteus* | ZALU.LNF | MSB:Mamm:37326 | F | 1978 | 32.95754 | -105.687 | 2587 | 0.070743 | 0.465981 | 0.50439 | 88 | 120 | 373 | 7140 | 251 | -71 | 322 | 178 | 118 | 182 | -12 | 502 | 110 | 13 | 77 | 329 | 49 | 253 | 84 |
| *Z. luteus* | ZALU.LNF | MSB:Mamm:41058 | F | 1979 | 32.95754 | -105.687 | 2587 | 0.072302 | 0.492836 | 0.520662 | 88 | 120 | 373 | 7140 | 251 | -71 | 322 | 178 | 118 | 182 | -12 | 502 | 110 | 13 | 77 | 329 | 49 | 253 | 84 |
| *Z. luteus* | ZALU.LNF | MSB:Mamm:41059 | M | 1979 | 32.95754 | -105.687 | 2587 | 0.075624 | 0.50618 | 0.557637 | 88 | 120 | 373 | 7140 | 251 | -71 | 322 | 178 | 118 | 182 | -12 | 502 | 110 | 13 | 77 | 329 | 49 | 253 | 84 |
| *Z. luteus* | ZALU.LNF | MSB:Mamm:41060 | F | 1979 | 32.95754 | -105.687 | 2587 | 0.070144 | 0.485917 | 0.552272 | 88 | 120 | 373 | 7140 | 251 | -71 | 322 | 178 | 118 | 182 | -12 | 502 | 110 | 13 | 77 | 329 | 49 | 253 | 84 |
| *Z. luteus* | ZALU.LNF | MSB:Mamm:41061 | F | 1979 | 32.95754 | -105.687 | 2587 | 0.069926 | 0.499935 | 0.565002 | 88 | 120 | 373 | 7140 | 251 | -71 | 322 | 178 | 118 | 182 | -12 | 502 | 110 | 13 | 77 | 329 | 49 | 253 | 84 |
| *Z. luteus* | ZALU.LNF | MSB:Mamm:41062 | F | 1979 | 32.95754 | -105.687 | 2587 | 0.080861 | 0.496246 | 0.534749 | 88 | 120 | 373 | 7140 | 251 | -71 | 322 | 178 | 118 | 182 | -12 | 502 | 110 | 13 | 77 | 329 | 49 | 253 | 84 |
| *Z. luteus* | ZALU.LNF | MSB:Mamm:41063 | M | 1979 | 32.95754 | -105.687 | 2587 | 0.070834 | 0.504215 | 0.556763 | 88 | 120 | 373 | 7140 | 251 | -71 | 322 | 178 | 118 | 182 | -12 | 502 | 110 | 13 | 77 | 329 | 49 | 253 | 84 |
| *Z. luteus* | ZALU.LNF | MSB:Mamm:41064 | F | 1979 | 32.95754 | -105.687 | 2587 | 0.070556 | 0.53926 | 0.55176 | 88 | 120 | 373 | 7140 | 251 | -71 | 322 | 178 | 118 | 182 | -12 | 502 | 110 | 13 | 77 | 329 | 49 | 253 | 84 |
| *Z. luteus* | ZALU.LNF | MSB:Mamm:41065 | M | 1979 | 32.95754 | -105.687 | 2587 | 0.07065 | 0.485994 | 0.56647 | 88 | 120 | 373 | 7140 | 251 | -71 | 322 | 178 | 118 | 182 | -12 | 502 | 110 | 13 | 77 | 329 | 49 | 253 | 84 |
| *Z. luteus* | ZALU.LNF | MSB:Mamm:41066 | F | 1979 | 32.95754 | -105.687 | 2587 | 0.070935 | 0.489627 | 0.53828 | 88 | 120 | 373 | 7140 | 251 | -71 | 322 | 178 | 118 | 182 | -12 | 502 | 110 | 13 | 77 | 329 | 49 | 253 | 84 |
| *Z. luteus* | ZALU.LNF | MSB:Mamm:37154 | M | 1977 | 32.95787 | -105.604 | 2480 | 0.080867 | 0.492333 | 0.565017 | 94 | 120 | 372 | 7174 | 258 | -66 | 324 | 184 | 80 | 189 | -6 | 623 | 136 | 16 | 79 | 406 | 61 | 325 | 87 |
| *Z. luteus* | ZALU.LNF | MSB:Mamm:37155 | M | 1977 | 32.95787 | -105.604 | 2480 | 0.072023 | 0.507667 | 0.562238 | 94 | 120 | 372 | 7174 | 258 | -66 | 324 | 184 | 80 | 189 | -6 | 623 | 136 | 16 | 79 | 406 | 61 | 325 | 87 |
| *Z. luteus* | ZALU.LNF | MSB:Mamm:61700 | F | 1988 | 32.9819 | -105.697 | 2647 | 0.071153 | 0.460964 | 0.507919 | 84 | 120 | 373 | 7123 | 247 | -75 | 322 | 174 | 114 | 178 | -15 | 599 | 140 | 15 | 85 | 418 | 55 | 320 | 90 |
| *Z. luteus* | ZALU.LNF | MSB:Mamm:61701 | F | 1988 | 32.9819 | -105.697 | 2647 | 0.078064 | 0.482933 | 0.531751 | 84 | 120 | 373 | 7123 | 247 | -75 | 322 | 174 | 114 | 178 | -15 | 599 | 140 | 15 | 85 | 418 | 55 | 320 | 90 |
| *Z. luteus* | ZALU.LNF | MSB:Mamm:61702 | F | 1988 | 32.9819 | -105.697 | 2647 | 0.068913 | 0.466747 | 0.509007 | 84 | 120 | 373 | 7123 | 247 | -75 | 322 | 174 | 114 | 178 | -15 | 599 | 140 | 15 | 85 | 418 | 55 | 320 | 90 |
| *Z. luteus* | ZALU.LNF | MSB:Mamm:61703 | F | 1988 | 33.00417 | -105.656 | 2482 | 0.075343 | 0.493244 | 0.581022 | 94 | 120 | 371 | 7181 | 258 | -66 | 324 | 184 | 124 | 189 | -6 | 495 | 109 | 13 | 78 | 325 | 51 | 255 | 76 |
| *Z. luteus* | ZALU.LNF | MSB:Mamm:61704 | F | 1988 | 33.00417 | -105.656 | 2482 | 0.075031 | 0.513287 | 0.578449 | 94 | 120 | 371 | 7181 | 258 | -66 | 324 | 184 | 124 | 189 | -6 | 495 | 109 | 13 | 78 | 325 | 51 | 255 | 76 |
| *Z. luteus* | ZALU.LNF | MSB:Mamm:36142 | F | 1977 | 33.01861 | -105.624 | 2374 | 0.073274 | 0.468006 | 0.533259 | 101 | 120 | 370 | 7218 | 265 | -60 | 325 | 191 | 87 | 196 | 0 | 460 | 98 | 13 | 75 | 293 | 50 | 230 | 73 |
| *Z. luteus* | ZALU.ASNF | MVZ:Mamm:56822 | M | 1932 | 33.6579 | -109.315 | 2760 | 0.07305 | 0.533195 | 0.56536 | 72 | 122 | 383 | 6986 | 239 | -80 | 319 | 165 | 90 | 167 | -21 | 622 | 120 | 16 | 60 | 347 | 52 | 236 | 147 |
| *Z. luteus* | ZALU.ASNF | MVZ:Mamm:61313 | M | 1933 | 33.6579 | -109.315 | 2760 | 0.072572 | 0.553283 | 0.62744 | 72 | 122 | 383 | 6986 | 239 | -80 | 319 | 165 | 90 | 167 | -21 | 622 | 120 | 16 | 60 | 347 | 52 | 236 | 147 |
| *Z. luteus* | ZALU.ASNF | MVZ:Mamm:61314 | F | 1933 | 33.6579 | -109.315 | 2760 | 0.072101 | 0.563587 | 0.598594 | 72 | 122 | 383 | 6986 | 239 | -80 | 319 | 165 | 90 | 167 | -21 | 622 | 120 | 16 | 60 | 347 | 52 | 236 | 147 |
| *Z. luteus* | ZALU.ASNF | MSB:Mamm:86344 | F | 1991 | 33.71919 | -109.443 | 2272 | 0.070632 | 0.461777 | 0.57288 | 101 | 122 | 378 | 7166 | 270 | -54 | 324 | 195 | 120 | 198 | 5 | 515 | 90 | 13 | 53 | 262 | 44 | 181 | 130 |
| *Z. luteus* | ZALU.ASNF | MSB:Mamm:89194 | F | 1991 | 33.74199 | -109.44 | 2352 | 0.078043 | 0.462339 | 0.517605 | 96 | 122 | 378 | 7154 | 265 | -58 | 323 | 190 | 115 | 193 | 0 | 562 | 98 | 15 | 53 | 285 | 50 | 197 | 140 |
| *Z. luteus* | ZALU.ASNF | MSB:Mamm:91675 | F | 1991 | 33.7707 | -109.439 | 2392 | 0.068169 | 0.475971 | 0.531133 | 94 | 122 | 378 | 7147 | 263 | -61 | 323 | 187 | 113 | 190 | -2 | 581 | 105 | 15 | 54 | 300 | 50 | 199 | 145 |
| *Z. luteus* | ZALU.ASNF | MSB:Mamm:181062 | F | 1974 | 33.77597 | -109.352 | 2381 | 0.10591 | 0.504304 | 0.519423 | 94 | 122 | 377 | 7154 | 263 | -60 | 323 | 188 | 113 | 191 | -2 | 543 | 96 | 16 | 53 | 280 | 51 | 196 | 123 |
| *Z. luteus* | ZALU.ASNF | MSB:Mamm:40949 | F | 1979 | 33.79 | -109.421 | 2448 | 0.076664 | 0.46613 | 0.568304 | 90 | 122 | 378 | 7137 | 259 | -64 | 323 | 184 | 109 | 187 | -5 | 581 | 108 | 15 | 55 | 306 | 49 | 199 | 143 |
| *Z. luteus* | ZALU.ASNF | MSB:Mamm:40950 | M | 1979 | 33.79 | -109.421 | 2448 | 0.080594 | 0.484849 | 0.532587 | 90 | 122 | 378 | 7137 | 259 | -64 | 323 | 184 | 109 | 187 | -5 | 581 | 108 | 15 | 55 | 306 | 49 | 199 | 143 |
| *Z. luteus* | ZALU.ASNF | MSB:Mamm:40951 | M | 1979 | 33.79 | -109.421 | 2448 | 0.080351 | 0.433075 | 0.558855 | 90 | 122 | 378 | 7137 | 259 | -64 | 323 | 184 | 109 | 187 | -5 | 581 | 108 | 15 | 55 | 306 | 49 | 199 | 143 |
| *Z. luteus* | ZALU.ASNF | MSB:Mamm:40952 | F | 1979 | 33.79 | -109.421 | 2448 | 0.068255 | 0.4577 | 0.554281 | 90 | 122 | 378 | 7137 | 259 | -64 | 323 | 184 | 109 | 187 | -5 | 581 | 108 | 15 | 55 | 306 | 49 | 199 | 143 |
| *Z. luteus* | ZALU.ASNF | MSB:Mamm:40953 | F | 1979 | 33.79 | -109.421 | 2448 | 0.070426 | 0.468945 | 0.509179 | 90 | 122 | 378 | 7137 | 259 | -64 | 323 | 184 | 109 | 187 | -5 | 581 | 108 | 15 | 55 | 306 | 49 | 199 | 143 |
| *Z. luteus* | ZALU.ASNF | MSB:Mamm:40954 | M | 1979 | 33.79 | -109.421 | 2448 | 0.087407 | 0.444118 | 0.512507 | 90 | 122 | 378 | 7137 | 259 | -64 | 323 | 184 | 109 | 187 | -5 | 581 | 108 | 15 | 55 | 306 | 49 | 199 | 143 |
| *Z. luteus* | ZALU.ASNF | MSB:Mamm:40955 | F | 1979 | 33.79 | -109.421 | 2448 | 0.072572 | 0.479447 | 0.548109 | 90 | 122 | 378 | 7137 | 259 | -64 | 323 | 184 | 109 | 187 | -5 | 581 | 108 | 15 | 55 | 306 | 49 | 199 | 143 |
| *Z. luteus* | ZALU.ASNF | MSB:Mamm:40956 | M | 1979 | 33.79 | -109.421 | 2448 | 0.08745 | 0.400586 | 0.555941 | 90 | 122 | 378 | 7137 | 259 | -64 | 323 | 184 | 109 | 187 | -5 | 581 | 108 | 15 | 55 | 306 | 49 | 199 | 143 |
| *Z. luteus* | ZALU.ASNF | MSB:Mamm:40994 | M | 1979 | 33.79 | -109.421 | 2448 | 0.06671 | 0.423126 | 0.547424 | 90 | 122 | 378 | 7137 | 259 | -64 | 323 | 184 | 109 | 187 | -5 | 581 | 108 | 15 | 55 | 306 | 49 | 199 | 143 |
| *Z. luteus* | ZALU.ASNF | MSB:Mamm:40995 | F | 1979 | 33.79 | -109.421 | 2448 | 0.073996 | 0.486559 | 0.530026 | 90 | 122 | 378 | 7137 | 259 | -64 | 323 | 184 | 109 | 187 | -5 | 581 | 108 | 15 | 55 | 306 | 49 | 199 | 143 |
| *Z. luteus* | ZALU.ASNF | MSB:Mamm:40996 | F | 1979 | 33.79 | -109.421 | 2448 | 0.070934 | 0.482515 | 0.549034 | 90 | 122 | 378 | 7137 | 259 | -64 | 323 | 184 | 109 | 187 | -5 | 581 | 108 | 15 | 55 | 306 | 49 | 199 | 143 |
| *Z. luteus* | ZALU.ASNF | MSB:Mamm:40997 | M | 1979 | 33.79 | -109.421 | 2448 | 0.074631 | 0.518389 | 0.57384 | 90 | 122 | 378 | 7137 | 259 | -64 | 323 | 184 | 109 | 187 | -5 | 581 | 108 | 15 | 55 | 306 | 49 | 199 | 143 |
| *Z. luteus* | ZALU.ASNF | MSB:Mamm:40998 | F | 1979 | 33.79 | -109.421 | 2448 | 0.070355 | 0.513735 | 0.527886 | 90 | 122 | 378 | 7137 | 259 | -64 | 323 | 184 | 109 | 187 | -5 | 581 | 108 | 15 | 55 | 306 | 49 | 199 | 143 |
| *Z. luteus* | ZALU.ASNF | MVZ:Mamm:61315 | M | 1933 | 33.7945 | -109.415 | 2442 | 0.070914 | 0.590607 | 0.566174 | 91 | 122 | 378 | 7143 | 260 | -64 | 323 | 184 | 109 | 187 | -5 | 576 | 107 | 15 | 56 | 304 | 49 | 199 | 140 |
| *Z. luteus* | ZALU.ASNF | MVZ:Mamm:61316 | M | 1933 | 33.7945 | -109.415 | 2442 | 0.069772 | 0.578323 | 0.575858 | 91 | 122 | 378 | 7143 | 260 | -64 | 323 | 184 | 109 | 187 | -5 | 576 | 107 | 15 | 56 | 304 | 49 | 199 | 140 |
| *Z. luteus* | ZALU.ASNF | MVZ:Mamm:61317 | F | 1933 | 33.7945 | -109.415 | 2442 | 0.072385 | 0.539183 | 0.565655 | 91 | 122 | 378 | 7143 | 260 | -64 | 323 | 184 | 109 | 187 | -5 | 576 | 107 | 15 | 56 | 304 | 49 | 199 | 140 |
| *Z. luteus* | ZALU.ASNF | MVZ:Mamm:61318 | F | 1933 | 33.7945 | -109.415 | 2442 | 0.071325 | 0.560575 | 0.591306 | 91 | 122 | 378 | 7143 | 260 | -64 | 323 | 184 | 109 | 187 | -5 | 576 | 107 | 15 | 56 | 304 | 49 | 199 | 140 |
| *Z. luteus* | ZALU.ASNF | MVZ:Mamm:61319 | M | 1933 | 33.7945 | -109.415 | 2442 | 0.070121 | 0.565747 | 0.569086 | 91 | 122 | 378 | 7143 | 260 | -64 | 323 | 184 | 109 | 187 | -5 | 576 | 107 | 15 | 56 | 304 | 49 | 199 | 140 |
| *Z. luteus* | ZALU.ASNF | MVZ:Mamm:61320 | M | 1933 | 33.7945 | -109.415 | 2442 | 0.071247 | 0.535508 | 0.605028 | 91 | 122 | 378 | 7143 | 260 | -64 | 323 | 184 | 109 | 187 | -5 | 576 | 107 | 15 | 56 | 304 | 49 | 199 | 140 |
| *Z. luteus* | ZALU.ASNF | MVZ:Mamm:61321 | M | 1933 | 33.7945 | -109.415 | 2442 | 0.071495 | 0.594743 | 0.510613 | 91 | 122 | 378 | 7143 | 260 | -64 | 323 | 184 | 109 | 187 | -5 | 576 | 107 | 15 | 56 | 304 | 49 | 199 | 140 |
| *Z. luteus* | ZALU.ASNF | MVZ:Mamm:61322 | M | 1933 | 33.7945 | -109.415 | 2442 | 0.07204 | 0.55408 | 0.56408 | 91 | 122 | 378 | 7143 | 260 | -64 | 323 | 184 | 109 | 187 | -5 | 576 | 107 | 15 | 56 | 304 | 49 | 199 | 140 |
| *Z. luteus* | ZALU.RIOGRAND | MSB:Mamm:36119 | F | 1976 | 33.8 | -106.867 | 1375 | 0.078193 | 0.45956 | 0.642105 | 164 | 122 | 347 | 8083 | 343 | -8 | 351 | 264 | 68 | 271 | 51 | 212 | 43 | 7 | 70 | 123 | 21 | 89 | 27 |
| *Z. luteus* | ZALU.RIOGRAND | MSB:Mamm:36143 | F | 1977 | 33.8 | -106.867 | 1375 | 0.071809 | 0.459814 | 0.606987 | 164 | 122 | 347 | 8083 | 343 | -8 | 351 | 264 | 68 | 271 | 51 | 212 | 43 | 7 | 70 | 123 | 21 | 89 | 27 |
| *Z. luteus* | ZALU.RIOGRAND | MSB:Mamm:41223 | M | 1979 | 33.80177 | -106.866 | 1376 | 0.07799 | 0.480812 | 0.614889 | 164 | 122 | 347 | 8083 | 343 | -8 | 351 | 264 | 68 | 271 | 51 | 212 | 43 | 7 | 70 | 123 | 21 | 89 | 27 |
| *Z. luteus* | ZALU.RIOGRAND | MSB:Mamm:41224 | F | 1979 | 33.80177 | -106.866 | 1376 | 0.076164 | 0.452216 | 0.562182 | 164 | 122 | 347 | 8083 | 343 | -8 | 351 | 264 | 68 | 271 | 51 | 212 | 43 | 7 | 70 | 123 | 21 | 89 | 27 |
| *Z. luteus* | ZALU.RIOGRAND | MSB:Mamm:41225 | M | 1979 | 33.80177 | -106.866 | 1376 | 0.073881 | 0.455626 | 0.590489 | 164 | 122 | 347 | 8083 | 343 | -8 | 351 | 264 | 68 | 271 | 51 | 212 | 43 | 7 | 70 | 123 | 21 | 89 | 27 |
| *Z. luteus* | ZALU.RIOGRAND | MSB:Mamm:41226 | F | 1979 | 33.80177 | -106.866 | 1376 | 0.092902 | 0.440462 | 0.587832 | 164 | 122 | 347 | 8083 | 343 | -8 | 351 | 264 | 68 | 271 | 51 | 212 | 43 | 7 | 70 | 123 | 21 | 89 | 27 |
| *Z. luteus* | ZALU.RIOGRAND | MSB:Mamm:41227 | M | 1979 | 33.80177 | -106.866 | 1376 | 0.071864 | 0.466284 | 0.623689 | 164 | 122 | 347 | 8083 | 343 | -8 | 351 | 264 | 68 | 271 | 51 | 212 | 43 | 7 | 70 | 123 | 21 | 89 | 27 |
| *Z. luteus* | ZALU.RIOGRAND | MSB:Mamm:41228 | F | 1979 | 33.80177 | -106.866 | 1376 | 0.077873 | 0.47463 | 0.599907 | 164 | 122 | 347 | 8083 | 343 | -8 | 351 | 264 | 68 | 271 | 51 | 212 | 43 | 7 | 70 | 123 | 21 | 89 | 27 |
| *Z. luteus* | ZALU.RIOGRAND | MSB:Mamm:41229 | F | 1979 | 33.80177 | -106.866 | 1376 | 0.072796 | 0.461791 | 0.575585 | 164 | 122 | 347 | 8083 | 343 | -8 | 351 | 264 | 68 | 271 | 51 | 212 | 43 | 7 | 70 | 123 | 21 | 89 | 27 |
| *Z. luteus* | ZALU.RIOGRAND | MSB:Mamm:41231 | F | 1979 | 33.80177 | -106.866 | 1376 | 0.076024 | 0.468017 | 0.633373 | 164 | 122 | 347 | 8083 | 343 | -8 | 351 | 264 | 68 | 271 | 51 | 212 | 43 | 7 | 70 | 123 | 21 | 89 | 27 |
| *Z. luteus* | ZALU.RIOGRAND | MSB:Mamm:41232 | F | 1979 | 33.80177 | -106.866 | 1376 | 0.075216 | 0.461827 | 0.616324 | 164 | 122 | 347 | 8083 | 343 | -8 | 351 | 264 | 68 | 271 | 51 | 212 | 43 | 7 | 70 | 123 | 21 | 89 | 27 |
| *Z. luteus* | ZALU.RIOGRAND | MSB:Mamm:41234 | F | 1979 | 33.80177 | -106.866 | 1376 | 0.076194 | 0.457422 | 0.602346 | 164 | 122 | 347 | 8083 | 343 | -8 | 351 | 264 | 68 | 271 | 51 | 212 | 43 | 7 | 70 | 123 | 21 | 89 | 27 |
| *Z. luteus* | ZALU.RIOGRAND | MSB:Mamm:41235 | F | 1979 | 33.80177 | -106.866 | 1376 | 0.09319 | 0.490929 | 0.631056 | 164 | 122 | 347 | 8083 | 343 | -8 | 351 | 264 | 68 | 271 | 51 | 212 | 43 | 7 | 70 | 123 | 21 | 89 | 27 |
| *Z. luteus* | ZALU.ASNF | MSB:Mamm:91627 | F | 1991 | 33.8862 | -109.484 | 2787 | 0.071858 | 0.454683 | 0.557109 | 69 | 122 | 379 | 7071 | 238 | -83 | 321 | 163 | 87 | 165 | -25 | 572 | 111 | 14 | 58 | 311 | 47 | 195 | 148 |
| *Z. luteus* | ZALU.ASNF | NA | F | 1972 | 33.95977 | -109.506 | 2834 | 0.077982 | 0.505202 | 0.576781 | 67 | 122 | 377 | 7102 | 236 | -87 | 322 | 161 | 84 | 163 | -28 | 495 | 97 | 13 | 59 | 275 | 43 | 178 | 128 |
| *Z. luteus* | ZALU.ASNF | NA | M | 1972 | 33.95977 | -109.506 | 2834 | 0.078154 | 0.540691 | 0.523581 | 67 | 122 | 377 | 7102 | 236 | -87 | 322 | 161 | 84 | 163 | -28 | 495 | 97 | 13 | 59 | 275 | 43 | 178 | 128 |
| *Z. luteus* | ZALU.ASNF | NA | M | 1972 | 33.95977 | -109.506 | 2834 | 0.078442 | 0.548533 | 0.55724 | 67 | 122 | 377 | 7102 | 236 | -87 | 322 | 161 | 84 | 163 | -28 | 495 | 97 | 13 | 59 | 275 | 43 | 178 | 128 |
| *Z. luteus* | ZALU.ASNF | NA | F | 1972 | 33.95977 | -109.506 | 2834 | 0.078415 | 0.525013 | 0.532189 | 67 | 122 | 377 | 7102 | 236 | -87 | 322 | 161 | 84 | 163 | -28 | 495 | 97 | 13 | 59 | 275 | 43 | 178 | 128 |
| *Z. luteus* | ZALU.ASNF | NA | F | 1972 | 33.95977 | -109.506 | 2834 | 0.07784 | 0.576011 | 0.571064 | 67 | 122 | 377 | 7102 | 236 | -87 | 322 | 161 | 84 | 163 | -28 | 495 | 97 | 13 | 59 | 275 | 43 | 178 | 128 |
| *Z. luteus* | ZALU.ASNF | NA | M | 1972 | 33.95977 | -109.506 | 2834 | 0.078717 | 0.55068 | 0.536933 | 67 | 122 | 377 | 7102 | 236 | -87 | 322 | 161 | 84 | 163 | -28 | 495 | 97 | 13 | 59 | 275 | 43 | 178 | 128 |
| *Z. luteus* | ZALU.ASNF | NA | M | 1968 | 33.95977 | -109.506 | 2834 | 0.07829 | 0.572514 | 0.574239 | 67 | 122 | 377 | 7102 | 236 | -87 | 322 | 161 | 84 | 163 | -28 | 495 | 97 | 13 | 59 | 275 | 43 | 178 | 128 |
| *Z. luteus* | ZALU.ASNF | MSB:Mamm:181060 | F | 1963 | 33.95977 | -109.506 | 2834 | 0.068914 | 0.488713 | 0.559341 | 67 | 122 | 377 | 7102 | 236 | -87 | 322 | 161 | 84 | 163 | -28 | 495 | 97 | 13 | 59 | 275 | 43 | 178 | 128 |
| *Z. luteus* | ZALU.ASNF | MSB:Mamm:181061 | F | 1963 | 33.95977 | -109.506 | 2834 | 0.084626 | 0.544865 | 0.541084 | 67 | 122 | 377 | 7102 | 236 | -87 | 322 | 161 | 84 | 163 | -28 | 495 | 97 | 13 | 59 | 275 | 43 | 178 | 128 |
| *Z. luteus* | ZALU.ASNF | MSB:Mamm:181058 | F | 1933 | 34.03787 | -109.845 | 1992 | 0.070078 | 0.554102 | 0.539681 | 117 | 122 | 368 | 7451 | 290 | -41 | 332 | 215 | 189 | 218 | 17 | 547 | 83 | 17 | 42 | 238 | 51 | 161 | 144 |
| *Z. luteus* | ZALU.ASNF | MSB:Mamm:181059 | M | 1933 | 34.03787 | -109.845 | 1992 | 0.070929 | 0.585538 | 0.526464 | 117 | 122 | 368 | 7451 | 290 | -41 | 332 | 215 | 189 | 218 | 17 | 547 | 83 | 17 | 42 | 238 | 51 | 161 | 144 |
| *Z. luteus* | ZALU.RIOGRAND | MSB:Mamm:62103 | M | 1987 | 34.66 | -106.737 | 1468 | 0.078884 | 0.42112 | 0.650024 | 152 | 122 | 341 | 8427 | 334 | -23 | 357 | 259 | 53 | 265 | 35 | 220 | 41 | 9 | 57 | 116 | 29 | 82 | 32 |
| *Z. luteus* | ZALU.RIOGRAND | MSB:Mamm:58368 | F | 1987 | 34.9352 | -106.687 | 1493 | 0.070337 | 0.422586 | 0.574907 | 148 | 122 | 339 | 8520 | 330 | -28 | 358 | 257 | 47 | 262 | 30 | 205 | 39 | 9 | 56 | 111 | 29 | 78 | 30 |
| *Z. luteus* | ZALU.SFNF | MSB:Mamm:62096 | M | 1989 | 35.7049 | -106.733 | 1789 | 0.090576 | 0.444452 | 0.573747 | 121 | 121 | 330 | 8556 | 309 | -58 | 367 | 233 | 19 | 236 | 3 | 265 | 51 | 12 | 55 | 145 | 39 | 102 | 45 |
| *Z. princeps* | ZAPR.SangreCristo | MSB:Mamm:66298 | F | 1990 | 35.72667 | -105.835 | 2596 | 0.079908 | 0.408748 | 0.513314 | 70 | 122 | 348 | 7973 | 251 | -101 | 351 | 177 | -21 | 177 | -39 | 489 | 84 | 24 | 47 | 245 | 76 | 193 | 76 |
| *Z. princeps* | ZAPR.SangreCristo | MSB:Mamm:41124 | M | 1979 | 35.76056 | -105.797 | 3386 | 0.077006 | 0.419227 | 0.54516 | 23 | 122 | 356 | 7678 | 201 | -143 | 344 | 128 | -63 | 128 | -81 | 568 | 100 | 25 | 52 | 298 | 79 | 298 | 81 |
| *Z. princeps* | ZAPR.SangreCristo | MSB:Mamm:41125 | F | 1979 | 35.76056 | -105.797 | 3386 | 0.075892 | 0.445962 | 0.543715 | 23 | 122 | 356 | 7678 | 201 | -143 | 344 | 128 | -63 | 128 | -81 | 568 | 100 | 25 | 52 | 298 | 79 | 298 | 81 |
| *Z. princeps* | ZAPR.SangreCristo | MSB:Mamm:41126 | F | 1979 | 35.76056 | -105.797 | 3386 | 0.076582 | 0.420213 | 0.545782 | 23 | 122 | 356 | 7678 | 201 | -143 | 344 | 128 | -63 | 128 | -81 | 568 | 100 | 25 | 52 | 298 | 79 | 298 | 81 |
| *Z. princeps* | ZAPR.SangreCristo | MSB:Mamm:41127 | F | 1979 | 35.76056 | -105.797 | 3386 | 0.076433 | 0.455481 | 0.58406 | 23 | 122 | 356 | 7678 | 201 | -143 | 344 | 128 | -63 | 128 | -81 | 568 | 100 | 25 | 52 | 298 | 79 | 298 | 81 |
| *Z. princeps* | ZAPR.SangreCristo | MSB:Mamm:41128 | F | 1979 | 35.76056 | -105.797 | 3386 | 0.071322 | 0.401536 | 0.494088 | 23 | 122 | 356 | 7678 | 201 | -143 | 344 | 128 | -63 | 128 | -81 | 568 | 100 | 25 | 52 | 298 | 79 | 298 | 81 |
| *Z. princeps* | ZAPR.SangreCristo | MSB:Mamm:41129 | M | 1979 | 35.76056 | -105.797 | 3386 | 0.074605 | 0.422391 | 0.546614 | 23 | 122 | 356 | 7678 | 201 | -143 | 344 | 128 | -63 | 128 | -81 | 568 | 100 | 25 | 52 | 298 | 79 | 298 | 81 |
| *Z. princeps* | ZAPR.SangreCristo | MSB:Mamm:41130 | M | 1979 | 35.76056 | -105.797 | 3386 | 0.074498 | 0.432834 | 0.530704 | 23 | 122 | 356 | 7678 | 201 | -143 | 344 | 128 | -63 | 128 | -81 | 568 | 100 | 25 | 52 | 298 | 79 | 298 | 81 |
| *Z. princeps* | ZAPR.SangreCristo | MSB:Mamm:41131 | M | 1979 | 35.76056 | -105.797 | 3386 | 0.074271 | 0.433875 | 0.528395 | 23 | 122 | 356 | 7678 | 201 | -143 | 344 | 128 | -63 | 128 | -81 | 568 | 100 | 25 | 52 | 298 | 79 | 298 | 81 |
| *Z. princeps* | ZAPR.SangreCristo | MSB:Mamm:41132 | F | 1979 | 35.76056 | -105.797 | 3386 | 0.074715 | 0.414575 | 0.567724 | 23 | 122 | 356 | 7678 | 201 | -143 | 344 | 128 | -63 | 128 | -81 | 568 | 100 | 25 | 52 | 298 | 79 | 298 | 81 |
| *Z. princeps* | ZAPR.SangreCristo | MSB:Mamm:41133 | M | 1979 | 35.76056 | -105.797 | 3386 | 0.074609 | 0.419428 | 0.482198 | 23 | 122 | 356 | 7678 | 201 | -143 | 344 | 128 | -63 | 128 | -81 | 568 | 100 | 25 | 52 | 298 | 79 | 298 | 81 |
| *Z. princeps* | ZAPR.SangreCristo | MSB:Mamm:41134 | F | 1979 | 35.76056 | -105.797 | 3386 | 0.074891 | 0.404974 | 0.500123 | 23 | 122 | 356 | 7678 | 201 | -143 | 344 | 128 | -63 | 128 | -81 | 568 | 100 | 25 | 52 | 298 | 79 | 298 | 81 |
| *Z. princeps* | ZAPR.SangreCristo | MSB:Mamm:41135 | F | 1979 | 35.76056 | -105.797 | 3386 | 0.070148 | 0.39846 | 0.492275 | 23 | 122 | 356 | 7678 | 201 | -143 | 344 | 128 | -63 | 128 | -81 | 568 | 100 | 25 | 52 | 298 | 79 | 298 | 81 |
| *Z. princeps* | ZAPR.SangreCristo | MSB:Mamm:37616 | M | 1978 | 35.7718 | -105.799 | 3279 | 0.07583 | 0.419821 | 0.555108 | 30 | 122 | 355 | 7715 | 208 | -137 | 345 | 134 | -57 | 134 | -75 | 567 | 99 | 25 | 51 | 294 | 79 | 294 | 83 |
| *Z. princeps* | ZAPR.SangreCristo | MSB:Mamm:53504 | M | 1979 | 35.79194 | -105.796 | 3302 | 0.07512 | 0.434674 | 0.553873 | 28 | 122 | 355 | 7714 | 206 | -139 | 345 | 133 | -59 | 133 | -76 | 561 | 98 | 25 | 50 | 288 | 79 | 288 | 83 |
| *Z. princeps* | ZAPR.SangreCristo | MSB:Mamm:293033 | M | 2016 | 35.80366 | -105.659 | 2636 | 0.074228 | 0.431464 | 0.513919 | 67 | 122 | 350 | 7910 | 246 | -103 | 350 | 173 | -24 | 173 | -41 | 386 | 75 | 15 | 61 | 221 | 49 | 182 | 55 |
| *Z. princeps* | ZAPR.SangreCristo | MSB:Mamm:293035 | M | 2016 | 35.80401 | -105.659 | 2636 | 0.074239 | 0.385762 | 0.485405 | 67 | 122 | 350 | 7910 | 246 | -103 | 350 | 173 | -24 | 173 | -41 | 386 | 75 | 15 | 61 | 221 | 49 | 182 | 55 |
| *Z. princeps* | ZAPR.SangreCristo | MSB:Mamm:293036 | F | 2016 | 35.80436 | -105.659 | 2636 | 0.082274 | 0.400209 | 0.53253 | 67 | 122 | 350 | 7910 | 246 | -103 | 350 | 173 | -24 | 173 | -41 | 386 | 75 | 15 | 61 | 221 | 49 | 182 | 55 |
| *Z. princeps* | ZAPR.SangreCristo | MSB:Mamm:293032 | M | 2016 | 35.80453 | -105.659 | 2636 | 0.079228 | 0.388128 | 0.503374 | 67 | 122 | 350 | 7910 | 246 | -103 | 350 | 173 | -24 | 173 | -41 | 386 | 75 | 15 | 61 | 221 | 49 | 182 | 55 |
| *Z. princeps* | ZAPR.SangreCristo | MSB:Mamm:293031 | F | 2016 | 35.80461 | -105.659 | 2636 | 0.08041 | 0.352936 | 0.4506 | 67 | 122 | 350 | 7910 | 246 | -103 | 350 | 173 | -24 | 173 | -41 | 386 | 75 | 15 | 61 | 221 | 49 | 182 | 55 |
| *Z. princeps* | ZAPR.SangreCristo | MSB:Mamm:322968 | M | 2017 | 35.8065 | -105.66 | 2636 | 0.077925 | 0.352164 | 0.512587 | 67 | 122 | 350 | 7910 | 246 | -103 | 350 | 173 | -24 | 173 | -41 | 386 | 75 | 15 | 61 | 221 | 49 | 182 | 55 |
| *Z. princeps* | ZAPR.SangreCristo | MSB:Mamm:322964 | F | 2017 | 35.80704 | -105.66 | 2636 | 0.07516 | 0.365286 | 0.531113 | 67 | 122 | 350 | 7910 | 246 | -103 | 350 | 173 | -24 | 173 | -41 | 386 | 75 | 15 | 61 | 221 | 49 | 182 | 55 |
| *Z. princeps* | ZAPR.SangreCristo | MSB:Mamm:322973 | M | 2017 | 35.80705 | -105.66 | 2636 | 0.088354 | 0.410857 | 0.517052 | 67 | 122 | 350 | 7910 | 246 | -103 | 350 | 173 | -24 | 173 | -41 | 386 | 75 | 15 | 61 | 221 | 49 | 182 | 55 |
| *Z. princeps* | ZAPR.SangreCristo | MSB:Mamm:322965 | M | 2017 | 35.80731 | -105.66 | 2636 | 0.080332 | 0.40768 | 0.516 | 67 | 122 | 350 | 7910 | 246 | -103 | 350 | 173 | -24 | 173 | -41 | 386 | 75 | 15 | 61 | 221 | 49 | 182 | 55 |
| *Z. princeps* | ZAPR.SangreCristo | MSB:Mamm:322974 | F | 2017 | 35.80731 | -105.66 | 2636 | 0.084411 | 0.382415 | 0.507344 | 67 | 122 | 350 | 7910 | 246 | -103 | 350 | 173 | -24 | 173 | -41 | 386 | 75 | 15 | 61 | 221 | 49 | 182 | 55 |
| *Z. princeps* | ZAPR.SangreCristo | MSB:Mamm:323010 | M | 2017 | 35.81237 | -105.663 | 2561 | 0.082289 | 0.408015 | 0.548791 | 71 | 122 | 349 | 7940 | 251 | -99 | 351 | 177 | -20 | 178 | -37 | 376 | 72 | 15 | 59 | 212 | 49 | 175 | 53 |
| *Z. princeps* | ZAPR.SangreCristo | MSB:Mamm:325479 | F | 2018 | 35.8124 | -105.663 | 2561 | 0.077704 | 0.340525 | 0.481504 | 71 | 122 | 349 | 7940 | 251 | -99 | 351 | 177 | -20 | 178 | -37 | 376 | 72 | 15 | 59 | 212 | 49 | 175 | 53 |
| *Z. princeps* | ZAPR.SangreCristo | MSB:Mamm:325355 | M | 2018 | 35.8124 | -105.662 | 2561 | 0.079507 | 0.36433 | 0.472204 | 71 | 122 | 349 | 7940 | 251 | -99 | 351 | 177 | -20 | 178 | -37 | 376 | 72 | 15 | 59 | 212 | 49 | 175 | 53 |
| *Z. princeps* | ZAPR.SangreCristo | MSB:Mamm:323934 | F | 2016 | 35.81243 | -105.662 | 2561 | 0.076232 | 0.390095 | 0.513771 | 71 | 122 | 349 | 7940 | 251 | -99 | 351 | 177 | -20 | 178 | -37 | 376 | 72 | 15 | 59 | 212 | 49 | 175 | 53 |
| *Z. princeps* | ZAPR.SangreCristo | MSB:Mamm:323012 | M | 2017 | 35.81249 | -105.663 | 2561 | 0.077285 | 0.397332 | 0.457625 | 71 | 122 | 349 | 7940 | 251 | -99 | 351 | 177 | -20 | 178 | -37 | 376 | 72 | 15 | 59 | 212 | 49 | 175 | 53 |
| *Z. princeps* | ZAPR.SangreCristo | MSB:Mamm:325332 | M | 2018 | 35.81254 | -105.663 | 2561 | 0.079473 | 0.349249 | 0.459135 | 71 | 122 | 349 | 7940 | 251 | -99 | 351 | 177 | -20 | 178 | -37 | 376 | 72 | 15 | 59 | 212 | 49 | 175 | 53 |
| *Z. princeps* | ZAPR.SangreCristo | MSB:Mamm:323011 | M | 2017 | 35.81255 | -105.663 | 2561 | 0.075871 | 0.350206 | 0.462129 | 71 | 122 | 349 | 7940 | 251 | -99 | 351 | 177 | -20 | 178 | -37 | 376 | 72 | 15 | 59 | 212 | 49 | 175 | 53 |
| *Z. princeps* | ZAPR.SangreCristo | MSB:Mamm:325480 | F | 2018 | 35.81264 | -105.663 | 2561 | 0.084485 | 0.403892 | 0.490851 | 71 | 122 | 349 | 7940 | 251 | -99 | 351 | 177 | -20 | 178 | -37 | 376 | 72 | 15 | 59 | 212 | 49 | 175 | 53 |
| *Z. princeps* | ZAPR.SangreCristo | MSB:Mamm:293102 | M | 2016 | 35.81592 | -105.679 | 2737 | 0.075157 | 0.420761 | 0.509828 | 60 | 122 | 351 | 7880 | 240 | -109 | 349 | 166 | -30 | 167 | -47 | 386 | 77 | 15 | 61 | 221 | 49 | 185 | 55 |
| *Z. princeps* | ZAPR.SangreCristo | MSB:Mamm:293104 | M | 2016 | 35.81595 | -105.68 | 2737 | 0.075523 | 0.415164 | 0.482513 | 60 | 122 | 351 | 7880 | 240 | -109 | 349 | 166 | -30 | 167 | -47 | 386 | 77 | 15 | 61 | 221 | 49 | 185 | 55 |
| *Z. princeps* | ZAPR.SangreCristo | MSB:Mamm:293099 | F | 2016 | 35.81601 | -105.681 | 2737 | 0.074725 | 0.400616 | 0.487761 | 60 | 122 | 351 | 7880 | 240 | -109 | 349 | 166 | -30 | 167 | -47 | 386 | 77 | 15 | 61 | 221 | 49 | 185 | 55 |
| *Z. princeps* | ZAPR.SangreCristo | MSB:Mamm:293109 | M | 2016 | 35.81603 | -105.681 | 2737 | 0.07544 | 0.481084 | 0.526521 | 60 | 122 | 351 | 7880 | 240 | -109 | 349 | 166 | -30 | 167 | -47 | 386 | 77 | 15 | 61 | 221 | 49 | 185 | 55 |
| *Z. princeps* | ZAPR.SangreCristo | MSB:Mamm:322969 | M | 2017 | 35.81606 | -105.68 | 2737 | 0.078681 | 0.396735 | 0.473212 | 60 | 122 | 351 | 7880 | 240 | -109 | 349 | 166 | -30 | 167 | -47 | 386 | 77 | 15 | 61 | 221 | 49 | 185 | 55 |
| *Z. princeps* | ZAPR.SangreCristo | MSB:Mamm:293110 | M | 2016 | 35.81608 | -105.68 | 2737 | 0.072935 | 0.402946 | 0.452537 | 60 | 122 | 351 | 7880 | 240 | -109 | 349 | 166 | -30 | 167 | -47 | 386 | 77 | 15 | 61 | 221 | 49 | 185 | 55 |
| *Z. princeps* | ZAPR.SangreCristo | MSB:Mamm:293103 | M | 2016 | 35.81623 | -105.681 | 2737 | 0.075911 | 0.416747 | 0.53238 | 60 | 122 | 351 | 7880 | 240 | -109 | 349 | 166 | -30 | 167 | -47 | 386 | 77 | 15 | 61 | 221 | 49 | 185 | 55 |
| *Z. princeps* | ZAPR.SangreCristo | MSB:Mamm:293105 | M | 2016 | 35.8163 | -105.684 | 2799 | 0.077781 | 0.436904 | 0.496523 | 57 | 122 | 351 | 7861 | 236 | -113 | 349 | 162 | -33 | 163 | -50 | 399 | 80 | 15 | 63 | 232 | 51 | 192 | 56 |
| *Z. princeps* | ZAPR.SangreCristo | MSB:Mamm:293107 | F | 2016 | 35.8163 | -105.685 | 2799 | 0.076758 | 0.430494 | 0.476638 | 57 | 122 | 351 | 7861 | 236 | -113 | 349 | 162 | -33 | 163 | -50 | 399 | 80 | 15 | 63 | 232 | 51 | 192 | 56 |
| *Z. princeps* | ZAPR.SangreCristo | MSB:Mamm:293083 | F | 2016 | 35.81647 | -105.682 | 2737 | 0.078238 | 0.442515 | 0.468073 | 60 | 122 | 351 | 7880 | 240 | -109 | 349 | 166 | -30 | 167 | -47 | 386 | 77 | 15 | 61 | 221 | 49 | 185 | 55 |
| *Z. princeps* | ZAPR.SangreCristo | MSB:Mamm:293200 | M | 2016 | 35.81649 | -105.682 | 2737 | 0.076319 | 0.400143 | 0.461643 | 60 | 122 | 351 | 7880 | 240 | -109 | 349 | 166 | -30 | 167 | -47 | 386 | 77 | 15 | 61 | 221 | 49 | 185 | 55 |
| *Z. princeps* | ZAPR.SangreCristo | MSB:Mamm:293201 | F | 2016 | 35.81649 | -105.682 | 2737 | 0.081838 | 0.409125 | 0.497168 | 60 | 122 | 351 | 7880 | 240 | -109 | 349 | 166 | -30 | 167 | -47 | 386 | 77 | 15 | 61 | 221 | 49 | 185 | 55 |
| *Z. princeps* | ZAPR.SangreCristo | MSB:Mamm:293202 | M | 2016 | 35.81649 | -105.682 | 2737 | 0.078848 | 0.405035 | 0.521078 | 60 | 122 | 351 | 7880 | 240 | -109 | 349 | 166 | -30 | 167 | -47 | 386 | 77 | 15 | 61 | 221 | 49 | 185 | 55 |
| *Z. princeps* | ZAPR.SangreCristo | MSB:Mamm:322967 | M | 2017 | 35.81653 | -105.682 | 2737 | 0.078371 | 0.349885 | 0.451555 | 61 | 122 | 350 | 7886 | 241 | -109 | 349 | 167 | -29 | 167 | -46 | 390 | 79 | 15 | 63 | 227 | 49 | 189 | 55 |
| *Z. princeps* | ZAPR.SangreCristo | MSB:Mamm:3233 | F | 1957 | 35.8202 | -105.661 | 2649 | 0.072364 | 0.391221 | 0.517575 | 66 | 122 | 350 | 7909 | 246 | -104 | 350 | 172 | -25 | 172 | -42 | 371 | 71 | 15 | 59 | 209 | 49 | 173 | 53 |
| *Z. princeps* | ZAPR.SangreCristo | MSB:Mamm:293053 | M | 2016 | 35.83082 | -105.659 | 2594 | 0.083695 | 0.431054 | 0.517285 | 69 | 122 | 349 | 7932 | 249 | -101 | 351 | 175 | -22 | 176 | -39 | 377 | 73 | 15 | 59 | 213 | 49 | 177 | 53 |
| *Z. princeps* | ZAPR.SangreCristo | MSB:Mamm:293054 | M | 2016 | 35.83089 | -105.659 | 2594 | 0.075485 | 0.412021 | 0.531787 | 69 | 122 | 349 | 7932 | 249 | -101 | 351 | 175 | -22 | 176 | -39 | 377 | 73 | 15 | 59 | 213 | 49 | 177 | 53 |
| *Z. princeps* | ZAPR.SangreCristo | MSB:Mamm:292994 | M | 2016 | 35.83153 | -105.66 | 2594 | 0.079728 | 0.425866 | 0.522905 | 69 | 122 | 349 | 7932 | 249 | -101 | 351 | 175 | -22 | 176 | -39 | 377 | 73 | 15 | 59 | 213 | 49 | 177 | 53 |
| *Z. princeps* | ZAPR.SangreCristo | MSB:Mamm:293057 | M | 2016 | 35.83364 | -105.661 | 2679 | 0.075327 | 0.422682 | 0.48981 | 64 | 122 | 350 | 7903 | 244 | -106 | 350 | 170 | -26 | 171 | -44 | 399 | 79 | 15 | 62 | 231 | 51 | 191 | 56 |
| *Z. luteus* | ZALU.SFNF | MSB:Mamm:56985 | M | 1985 | 35.8489 | -106.768 | 2370 | 0.074395 | 0.499687 | 0.56122 | 85 | 121 | 334 | 8332 | 271 | -91 | 363 | 196 | -15 | 198 | -30 | 375 | 66 | 20 | 47 | 189 | 64 | 136 | 70 |
| *Z. luteus* | ZALU.SFNF | MSB:Mamm:62097 | M | 1989 | 35.8776 | -106.839 | 2765 | 0.075116 | 0.511549 | 0.561053 | 62 | 121 | 338 | 8180 | 247 | -112 | 359 | 172 | 140 | 172 | -50 | 478 | 85 | 26 | 47 | 244 | 82 | 175 | 93 |
| *Z. luteus* | ZALU.SFNF | MSB:Mamm:62098 | F | 1989 | 35.8776 | -106.839 | 2765 | 0.072943 | 0.472308 | 0.559331 | 62 | 121 | 338 | 8180 | 247 | -112 | 359 | 172 | 140 | 172 | -50 | 478 | 85 | 26 | 47 | 244 | 82 | 175 | 93 |
| *Z. luteus* | ZALU.SFNF | MSB:Mamm:56984 | F | 1985 | 35.8776 | -106.715 | 2466 | 0.082747 | 0.470046 | 0.541357 | 79 | 121 | 335 | 8279 | 265 | -97 | 362 | 189 | -21 | 190 | -35 | 419 | 71 | 23 | 43 | 202 | 73 | 146 | 79 |
| *Z. luteus* | ZALU.SFNF | MSB:Mamm:56979 | F | 1985 | 35.892 | -106.715 | 2467 | 0.084927 | 0.474334 | 0.553119 | 79 | 121 | 335 | 8281 | 265 | -98 | 362 | 189 | -21 | 190 | -35 | 376 | 67 | 20 | 48 | 192 | 63 | 139 | 67 |
| *Z. luteus* | ZALU.SFNF | MSB:Mamm:56980 | F | 1985 | 35.892 | -106.715 | 2467 | 0.079833 | 0.454076 | 0.504831 | 79 | 121 | 335 | 8281 | 265 | -98 | 362 | 189 | -21 | 190 | -35 | 376 | 67 | 20 | 48 | 192 | 63 | 139 | 67 |
| *Z. luteus* | ZALU.SFNF | MSB:Mamm:56981 | M | 1985 | 35.892 | -106.715 | 2467 | 0.119021 | 0.437259 | 0.571803 | 79 | 121 | 335 | 8281 | 265 | -98 | 362 | 189 | -21 | 190 | -35 | 376 | 67 | 20 | 48 | 192 | 63 | 139 | 67 |
| *Z. luteus* | ZALU.SFNF | MSB:Mamm:56982 | F | 1985 | 35.892 | -106.715 | 2467 | 0.084171 | 0.482187 | 0.548408 | 79 | 121 | 335 | 8281 | 265 | -98 | 362 | 189 | -21 | 190 | -35 | 376 | 67 | 20 | 48 | 192 | 63 | 139 | 67 |
| *Z. luteus* | ZALU.SFNF | MSB:Mamm:56983 | M | 1985 | 35.892 | -106.715 | 2467 | 0.075414 | 0.465828 | 0.538318 | 79 | 121 | 335 | 8281 | 265 | -98 | 362 | 189 | -21 | 190 | -35 | 376 | 67 | 20 | 48 | 192 | 63 | 139 | 67 |
| *Z. princeps* | ZAPR.SangreCristo | MSB:Mamm:36131 | F | 1976 | 35.90152 | -105.346 | 2256 | 0.078648 | 0.412876 | 0.547514 | 89 | 122 | 350 | 7950 | 269 | -81 | 350 | 195 | -3 | 196 | -20 | 460 | 85 | 17 | 59 | 255 | 55 | 216 | 57 |
| *Z. princeps* | ZAPR.SangreCristo | MSB:Mamm:36133 | F | 1976 | 35.90152 | -105.346 | 2256 | 0.074443 | 0.405238 | 0.455047 | 89 | 122 | 350 | 7950 | 269 | -81 | 350 | 195 | -3 | 196 | -20 | 460 | 85 | 17 | 59 | 255 | 55 | 216 | 57 |
| *Z. princeps* | ZAPR.SangreCristo | MSB:Mamm:36139 | F | 1976 | 35.90152 | -105.346 | 2256 | 0.074098 | 0.416561 | 0.51266 | 89 | 122 | 350 | 7950 | 269 | -81 | 350 | 195 | -3 | 196 | -20 | 460 | 85 | 17 | 59 | 255 | 55 | 216 | 57 |
| *Z. luteus* | ZALU.SFNF | MSB:Mamm:56993 | F | 1985 | 35.9208 | -106.698 | 2565 | 0.074977 | 0.515247 | 0.594978 | 72 | 122 | 336 | 8239 | 258 | -104 | 362 | 182 | -27 | 183 | -41 | 426 | 74 | 23 | 45 | 211 | 73 | 152 | 79 |
| *Z. luteus* | ZALU.SFNF | MSB:Mamm:56994 | F | 1985 | 35.9208 | -106.698 | 2565 | 0.07758 | 0.48943 | 0.607589 | 72 | 122 | 336 | 8239 | 258 | -104 | 362 | 182 | -27 | 183 | -41 | 426 | 74 | 23 | 45 | 211 | 73 | 152 | 79 |
| *Z. luteus* | ZALU.SFNF | MSB:Mamm:56986 | F | 1985 | 35.9352 | -106.786 | 2452 | 0.078369 | 0.496279 | 0.536097 | 79 | 121 | 334 | 8303 | 266 | -97 | 363 | 190 | 159 | 191 | -35 | 355 | 62 | 20 | 45 | 177 | 62 | 126 | 69 |
| *Z. luteus* | ZALU.SFNF | MSB:Mamm:56991 | M | 1985 | 35.9496 | -106.645 | 2629 | 0.072174 | 0.486758 | 0.541658 | 68 | 122 | 337 | 8203 | 253 | -108 | 361 | 178 | -31 | 179 | -45 | 470 | 80 | 26 | 42 | 227 | 82 | 163 | 88 |
| *Z. luteus* | ZALU.SFNF | MSB:Mamm:56992 | F | 1985 | 35.9496 | -106.645 | 2629 | 0.084027 | 0.469343 | 0.577832 | 68 | 122 | 337 | 8203 | 253 | -108 | 361 | 178 | -31 | 179 | -45 | 470 | 80 | 26 | 42 | 227 | 82 | 163 | 88 |
| *Z. luteus* | ZALU.SFNF | MSB:Mamm:62101 | M | 1989 | 35.9496 | -106.68 | 2671 | 0.070168 | 0.512134 | 0.547498 | 66 | 122 | 337 | 8191 | 251 | -110 | 361 | 175 | -28 | 176 | -47 | 455 | 79 | 25 | 45 | 226 | 78 | 163 | 84 |
| *Z. luteus* | ZALU.SFNF | MSB:Mamm:62102 | F | 1989 | 35.964 | -106.786 | 2442 | 0.074684 | 0.50519 | 0.546731 | 80 | 121 | 334 | 8310 | 266 | -97 | 364 | 190 | 159 | 192 | -35 | 342 | 60 | 19 | 45 | 171 | 59 | 121 | 66 |
| *Z. luteus* | ZALU.SFNF | MSB:Mamm:67525 | F | 1985 | 35.9784 | -106.786 | 2498 | 0.069636 | 0.48361 | 0.539451 | 76 | 121 | 334 | 8286 | 263 | -101 | 363 | 187 | 155 | 188 | -38 | 352 | 62 | 20 | 46 | 178 | 62 | 128 | 67 |
| *Z. luteus* | ZALU.SFNF | MSB:Mamm:56987 | M | 1985 | 35.9927 | -106.716 | 2585 | 0.07581 | 0.482417 | 0.56649 | 70 | 122 | 335 | 8237 | 256 | -106 | 363 | 181 | -24 | 182 | -43 | 421 | 72 | 23 | 44 | 207 | 70 | 152 | 75 |
| *Z. luteus* | ZALU.SFNF | MSB:Mamm:56988 | F | 1985 | 35.9927 | -106.716 | 2585 | 0.072736 | 0.479549 | 0.569975 | 70 | 122 | 335 | 8237 | 256 | -106 | 363 | 181 | -24 | 182 | -43 | 421 | 72 | 23 | 44 | 207 | 70 | 152 | 75 |
| *Z. luteus* | ZALU.SFNF | MSB:Mamm:56989 | M | 1985 | 35.9927 | -106.716 | 2585 | 0.084876 | 0.478411 | 0.570621 | 70 | 122 | 335 | 8237 | 256 | -106 | 363 | 181 | -24 | 182 | -43 | 421 | 72 | 23 | 44 | 207 | 70 | 152 | 75 |
| *Z. luteus* | ZALU.SFNF | MSB:Mamm:56990 | F | 1985 | 35.9927 | -106.716 | 2585 | 0.077001 | 0.502992 | 0.603632 | 70 | 122 | 335 | 8237 | 256 | -106 | 363 | 181 | -24 | 182 | -43 | 421 | 72 | 23 | 44 | 207 | 70 | 152 | 75 |
| *Z. luteus* | ZALU.ESPANOLA | MSB:Mamm:58371 | M | 1987 | 36.0382 | -106.063 | 1723 | 0.073391 | 0.47264 | 0.59522 | 117 | 123 | 334 | 8422 | 305 | -64 | 369 | 228 | 16 | 230 | 1 | 247 | 47 | 11 | 53 | 133 | 35 | 95 | 38 |
| *Z. princeps* | ZAPR.CNF | MVZ:Mamm:116582 | M | 1952 | 36.0397 | -105.687 | 2873 | 0.076293 | 0.457367 | 0.520489 | 51 | 123 | 349 | 7879 | 232 | -121 | 353 | 157 | -39 | 157 | -57 | 464 | 75 | 25 | 39 | 216 | 75 | 216 | 79 |
| *Z. princeps* | ZAPR.CNF | MVZ:Mamm:116583 | F | 1952 | 36.0397 | -105.687 | 2873 | 0.077514 | 0.484866 | 0.560446 | 51 | 123 | 349 | 7879 | 232 | -121 | 353 | 157 | -39 | 157 | -57 | 464 | 75 | 25 | 39 | 216 | 75 | 216 | 79 |
| *Z. princeps* | ZAPR.CNF | MVZ:Mamm:116584 | M | 1952 | 36.0397 | -105.687 | 2873 | 0.076981 | 0.447687 | 0.567526 | 51 | 123 | 349 | 7879 | 232 | -121 | 353 | 157 | -39 | 157 | -57 | 464 | 75 | 25 | 39 | 216 | 75 | 216 | 79 |
| *Z. princeps* | ZAPR.SangreCristo | MVZ:Mamm:116585 | F | 1952 | 36.0397 | -105.687 | 2873 | 0.076149 | 0.47236 | 0.522253 | 51 | 123 | 349 | 7879 | 232 | -121 | 353 | 157 | -39 | 157 | -57 | 464 | 75 | 25 | 39 | 216 | 75 | 216 | 79 |
| *Z. princeps* | ZAPR.CNF | MVZ:Mamm:116586 | NA | 1952 | 36.0397 | -105.687 | 2873 | 0.078033 | 0.474848 | 0.560723 | 51 | 123 | 349 | 7879 | 232 | -121 | 353 | 157 | -39 | 157 | -57 | 464 | 75 | 25 | 39 | 216 | 75 | 216 | 79 |
| *Z. princeps* | ZAPR.CNF | MVZ:Mamm:116587 | M | 1952 | 36.0397 | -105.687 | 2873 | 0.07645 | 0.468349 | 0.553537 | 51 | 123 | 349 | 7879 | 232 | -121 | 353 | 157 | -39 | 157 | -57 | 464 | 75 | 25 | 39 | 216 | 75 | 216 | 79 |
| *Z. luteus* | ZALU.ESPANOLA | MSB:Mamm:58370 | M | 1987 | 36.0527 | -106.08 | 1720 | 0.071915 | 0.439512 | 0.573243 | 117 | 123 | 334 | 8431 | 305 | -64 | 369 | 228 | 16 | 230 | 1 | 235 | 45 | 11 | 53 | 127 | 34 | 90 | 36 |
| *Z. princeps* | ZAPR.SangreCristo | MVZ:Mamm:116588 | F | 1952 | 36.05844 | -105.823 | 2378 | 0.076089 | 0.432567 | 0.535399 | 79 | 123 | 343 | 8107 | 263 | -97 | 360 | 187 | -14 | 188 | -32 | 413 | 73 | 21 | 47 | 209 | 65 | 156 | 67 |
| *Z. luteus* | ZALU.ESPANOLA | MSB:Mamm:58369 | M | 1987 | 36.0954 | -106.144 | 1780 | 0.074808 | 0.437215 | 0.616663 | 113 | 123 | 333 | 8431 | 301 | -69 | 370 | 225 | 12 | 227 | -3 | 229 | 43 | 11 | 50 | 120 | 34 | 83 | 36 |
| *Z. princeps* | ZAPR.SangreCristo | MSB:Mamm:35653 | M | 1977 | 36.13721 | -105.475 | 2791 | 0.075376 | 0.442387 | 0.544637 | 55 | 123 | 351 | 7848 | 235 | -116 | 351 | 161 | -40 | 161 | -52 | 430 | 74 | 21 | 48 | 219 | 64 | 219 | 66 |
| *Z. princeps* | ZAPR.SangreCristo | MSB:Mamm:4943 | M | 1958 | 36.1397 | -105.457 | 2867 | 0.071864 | 0.535074 | 0.558785 | 50 | 123 | 351 | 7817 | 231 | -120 | 351 | 156 | -44 | 156 | -56 | 500 | 83 | 26 | 44 | 244 | 78 | 244 | 78 |
| *Z. princeps* | ZAPR.SangreCristo | MSB:Mamm:4944 | F | 1958 | 36.1397 | -105.457 | 2867 | 0.073454 | 0.446012 | 0.531351 | 50 | 123 | 351 | 7817 | 231 | -120 | 351 | 156 | -44 | 156 | -56 | 500 | 83 | 26 | 44 | 244 | 78 | 244 | 78 |
| *Z. princeps* | ZAPR.SangreCristo | MSB:Mamm:4945 | M | 1958 | 36.1397 | -105.457 | 2867 | 0.072417 | 0.400331 | 0.478212 | 50 | 123 | 351 | 7817 | 231 | -120 | 351 | 156 | -44 | 156 | -56 | 500 | 83 | 26 | 44 | 244 | 78 | 244 | 78 |
| *Z. princeps* | ZAPR.SangreCristo | MSB:Mamm:41242 | M | 1979 | 36.14967 | -105.456 | 2824 | 0.07319 | 0.417536 | 0.50164 | 53 | 123 | 351 | 7835 | 234 | -118 | 351 | 159 | -41 | 159 | -53 | 508 | 90 | 24 | 51 | 265 | 72 | 265 | 72 |
| *Z. princeps* | ZAPR.SangreCristo | MSB:Mamm:41243 | M | 1979 | 36.14967 | -105.456 | 2824 | 0.067294 | 0.420528 | 0.470649 | 53 | 123 | 351 | 7835 | 234 | -118 | 351 | 159 | -41 | 159 | -53 | 508 | 90 | 24 | 51 | 265 | 72 | 265 | 72 |
| *Z. princeps* | ZAPR.SangreCristo | MSB:Mamm:41245 | F | 1979 | 36.14967 | -105.456 | 2824 | 0.073987 | 0.412015 | 0.521889 | 53 | 123 | 351 | 7835 | 234 | -118 | 351 | 159 | -41 | 159 | -53 | 508 | 90 | 24 | 51 | 265 | 72 | 265 | 72 |
| *Z. princeps* | ZAPR.SangreCristo | MSB:Mamm:41246 | F | 1979 | 36.14967 | -105.456 | 2824 | 0.076454 | 0.441118 | 0.537072 | 53 | 123 | 351 | 7835 | 234 | -118 | 351 | 159 | -41 | 159 | -53 | 508 | 90 | 24 | 51 | 265 | 72 | 265 | 72 |
| *Z. princeps* | ZAPR.SangreCristo | MSB:Mamm:41247 | M | 1979 | 36.14967 | -105.456 | 2824 | 0.070882 | 0.419326 | 0.514814 | 53 | 123 | 351 | 7835 | 234 | -118 | 351 | 159 | -41 | 159 | -53 | 508 | 90 | 24 | 51 | 265 | 72 | 265 | 72 |
| *Z. princeps* | ZAPR.SangreCristo | MSB:Mamm:41248 | M | 1979 | 36.14967 | -105.456 | 2824 | 0.072676 | 0.433806 | 0.518011 | 53 | 123 | 351 | 7835 | 234 | -118 | 351 | 159 | -41 | 159 | -53 | 508 | 90 | 24 | 51 | 265 | 72 | 265 | 72 |
| *Z. princeps* | ZAPR.SangreCristo | MSB:Mamm:272908 | M | 1994 | 36.17407 | -105.444 | 2923 | 0.077133 | 0.386788 | 0.570048 | 47 | 123 | 352 | 7800 | 227 | -123 | 351 | 153 | -47 | 153 | -59 | 555 | 104 | 24 | 57 | 308 | 72 | 308 | 72 |
| *Z. princeps* | ZAPR.SangreCristo | MSB:Mamm:272909 | F | 1994 | 36.17407 | -105.444 | 2923 | 0.076786 | 0.325879 | 0.582964 | 47 | 123 | 352 | 7800 | 227 | -123 | 351 | 153 | -47 | 153 | -59 | 555 | 104 | 24 | 57 | 308 | 72 | 308 | 72 |
| *Z. princeps* | ZAPR.SangreCristo | MSB:Mamm:272932 | F | 1994 | 36.17407 | -105.444 | 2923 | 0.078234 | 0.347887 | 0.55654 | 47 | 123 | 352 | 7800 | 227 | -123 | 351 | 153 | -47 | 153 | -59 | 555 | 104 | 24 | 57 | 308 | 72 | 308 | 72 |
| *Z. princeps* | ZAPR.SangreCristo | MSB:Mamm:272938 | F | 1994 | 36.17407 | -105.444 | 2923 | 0.076451 | 0.353137 | 0.520149 | 47 | 123 | 352 | 7800 | 227 | -123 | 351 | 153 | -47 | 153 | -59 | 555 | 104 | 24 | 57 | 308 | 72 | 308 | 72 |
| *Z. princeps* | ZAPR.SangreCristo | MSB:Mamm:273835 | M | 2000 | 36.23842 | -105.235 | 2525 | 0.0749 | 0.347899 | 0.551382 | 70 | 123 | 351 | 7870 | 251 | -100 | 351 | 176 | -36 | 176 | -36 | 421 | 78 | 16 | 60 | 234 | 48 | 196 | 48 |
| *Z. princeps* | ZAPR.SangreCristo | MSB:Mamm:273837 | F | 2000 | 36.23842 | -105.235 | 2525 | 0.073735 | 0.302223 | 0.520673 | 70 | 123 | 351 | 7870 | 251 | -100 | 351 | 176 | -36 | 176 | -36 | 421 | 78 | 16 | 60 | 234 | 48 | 196 | 48 |
| *Z. princeps* | ZAPR.SangreCristo | MSB:Mamm:335937 | M | 1990 | 36.24702 | -105.409 | 3374 | 0.069055 | 0.511764 | 0.575601 | 20 | 124 | 356 | 7648 | 199 | -148 | 347 | 125 | -72 | 125 | -83 | 588 | 95 | 30 | 43 | 279 | 90 | 279 | 90 |
| *Z. princeps* | ZAPR.SangreCristo | MSB:Mamm:335938 | F | 1990 | 36.24702 | -105.409 | 3374 | 0.067641 | 0.515594 | 0.53799 | 20 | 124 | 356 | 7648 | 199 | -148 | 347 | 125 | -72 | 125 | -83 | 588 | 95 | 30 | 43 | 279 | 90 | 279 | 90 |
| *Z. princeps* | ZAPR.SangreCristo | MSB:Mamm:43506 | F | 1980 | 36.24702 | -105.409 | 3374 | 0.074129 | 0.437042 | 0.568473 | 20 | 124 | 356 | 7648 | 199 | -148 | 347 | 125 | -72 | 125 | -83 | 588 | 95 | 30 | 43 | 279 | 90 | 279 | 90 |
| *Z. princeps* | ZAPR.SangreCristo | MSB:Mamm:43507 | F | 1980 | 36.24702 | -105.409 | 3374 | 0.071504 | 0.392132 | 0.55781 | 20 | 124 | 356 | 7648 | 199 | -148 | 347 | 125 | -72 | 125 | -83 | 588 | 95 | 30 | 43 | 279 | 90 | 279 | 90 |
| *Z. princeps* | ZAPR.SangreCristo | MSB:Mamm:43508 | F | 1980 | 36.24702 | -105.409 | 3374 | 0.069856 | 0.378009 | 0.527418 | 20 | 124 | 356 | 7648 | 199 | -148 | 347 | 125 | -72 | 125 | -83 | 588 | 95 | 30 | 43 | 279 | 90 | 279 | 90 |
| *Z. princeps* | ZAPR.SangreCristo | MSB:Mamm:43516 | M | 1980 | 36.24702 | -105.409 | 3374 | 0.07012 | 0.368822 | 0.516177 | 20 | 124 | 356 | 7648 | 199 | -148 | 347 | 125 | -72 | 125 | -83 | 588 | 95 | 30 | 43 | 279 | 90 | 279 | 90 |
| *Z. princeps* | ZAPR.SangreCristo | MSB:Mamm:43517 | M | 1980 | 36.24702 | -105.409 | 3374 | 0.073689 | 0.418309 | 0.576324 | 20 | 124 | 356 | 7648 | 199 | -148 | 347 | 125 | -72 | 125 | -83 | 588 | 95 | 30 | 43 | 279 | 90 | 279 | 90 |
| *Z. princeps* | ZAPR.SangreCristo | MSB:Mamm:43518 | M | 1980 | 36.24702 | -105.409 | 3374 | 0.071891 | 0.360651 | 0.584838 | 20 | 124 | 356 | 7648 | 199 | -148 | 347 | 125 | -72 | 125 | -83 | 588 | 95 | 30 | 43 | 279 | 90 | 279 | 90 |
| *Z. princeps* | ZAPR.SangreCristo | MSB:Mamm:43519 | M | 1980 | 36.24702 | -105.409 | 3374 | 0.072333 | 0.393694 | 0.553329 | 20 | 124 | 356 | 7648 | 199 | -148 | 347 | 125 | -72 | 125 | -83 | 588 | 95 | 30 | 43 | 279 | 90 | 279 | 90 |
| *Z. princeps* | ZAPR.SangreCristo | MSB:Mamm:43520 | F | 1980 | 36.24702 | -105.409 | 3374 | 0.072999 | 0.366969 | 0.580211 | 20 | 124 | 356 | 7648 | 199 | -148 | 347 | 125 | -72 | 125 | -83 | 588 | 95 | 30 | 43 | 279 | 90 | 279 | 90 |
| *Z. luteus* | ZALU.NENM | MSB:Mamm:325193 | M | 2018 | 36.3695 | -104.944 | 2020 | 0.069954 | 0.489024 | 0.541434 | 99 | 124 | 350 | 7978 | 281 | -72 | 353 | 206 | -9 | 206 | -9 | 362 | 67 | 10 | 63 | 196 | 32 | 164 | 32 |
| *Z. princeps* | ZAPR.NENM | MSB:Mamm:325873 | M | 2018 | 36.5374 | -105.149 | 2411 | 0.080476 | 0.403461 | 0.603296 | 74 | 125 | 350 | 7942 | 257 | -99 | 356 | 181 | -33 | 181 | -33 | 353 | 61 | 14 | 52 | 180 | 44 | 180 | 44 |
| *Z. princeps* | ZAPR.CNF | MSB:Mamm:37670 | F | 1977 | 36.55472 | -106.332 | 3034 | 0.072574 | 0.371479 | 0.549106 | 36 | 125 | 342 | 8020 | 222 | -145 | 367 | 145 | 109 | 145 | -74 | 538 | 82 | 29 | 33 | 232 | 96 | 232 | 113 |
| *Z. princeps* | ZAPR.CNF | MSB:Mamm:54006 | M | 1980 | 36.55472 | -106.332 | 3034 | 0.076041 | 0.44449 | 0.56442 | 36 | 125 | 342 | 8020 | 222 | -145 | 367 | 145 | 109 | 145 | -74 | 538 | 82 | 29 | 33 | 232 | 96 | 232 | 113 |
| *Z. princeps* | ZAPR.CNF | MSB:Mamm:54007 | M | 1980 | 36.55472 | -106.332 | 3034 | 0.073022 | 0.41803 | 0.531646 | 36 | 125 | 342 | 8020 | 222 | -145 | 367 | 145 | 109 | 145 | -74 | 538 | 82 | 29 | 33 | 232 | 96 | 232 | 113 |
| *Z. princeps* | ZAPR.CNF | MSB:Mamm:54008 | F | 1980 | 36.55472 | -106.332 | 3034 | 0.072659 | 0.405089 | 0.479946 | 36 | 125 | 342 | 8020 | 222 | -145 | 367 | 145 | 109 | 145 | -74 | 538 | 82 | 29 | 33 | 232 | 96 | 232 | 113 |
| *Z. princeps* | ZAPR.CNF | MSB:Mamm:54009 | F | 1980 | 36.55472 | -106.332 | 3034 | 0.073418 | 0.414826 | 0.536301 | 36 | 125 | 342 | 8020 | 222 | -145 | 367 | 145 | 109 | 145 | -74 | 538 | 82 | 29 | 33 | 232 | 96 | 232 | 113 |
| *Z. princeps* | ZAPR.SangreCristo | MSB:Mamm:41330 | M | 1979 | 36.55831 | -105.526 | 2661 | 0.078025 | 0.419718 | 0.511379 | 63 | 126 | 346 | 8053 | 249 | -115 | 364 | 172 | -34 | 172 | -46 | 428 | 70 | 21 | 46 | 210 | 63 | 210 | 63 |
| *Z. princeps* | ZAPR.SangreCristo | MSB:Mamm:41331 | M | 1979 | 36.55831 | -105.526 | 2661 | 0.076135 | 0.458784 | 0.511054 | 63 | 126 | 346 | 8053 | 249 | -115 | 364 | 172 | -34 | 172 | -46 | 428 | 70 | 21 | 46 | 210 | 63 | 210 | 63 |
| *Z. princeps* | ZAPR.CNF | MSB:Mamm:38009 | F | 1977 | 36.56111 | -106.329 | 3099 | 0.07623 | 0.412742 | 0.523815 | 32 | 126 | 343 | 7997 | 218 | -148 | 366 | 141 | 105 | 141 | -77 | 545 | 83 | 29 | 33 | 235 | 95 | 235 | 119 |
| *Z. princeps* | ZAPR.SangreCristo | MSB:Mamm:41332 | M | 1979 | 36.5656 | -105.517 | 2909 | 0.075926 | 0.45746 | 0.518784 | 43 | 126 | 349 | 7927 | 228 | -133 | 361 | 152 | -52 | 152 | -64 | 480 | 76 | 25 | 42 | 226 | 76 | 226 | 77 |
| *Z. princeps* | ZAPR.SangreCristo | MSB:Mamm:41267 | M | 1979 | 36.6025 | -105.486 | 3059 | 0.07433 | 0.430808 | 0.551386 | 35 | 126 | 350 | 7882 | 219 | -141 | 360 | 143 | -71 | 143 | -71 | 443 | 77 | 20 | 51 | 225 | 60 | 225 | 60 |
| *Z. princeps* | ZAPR.SangreCristo | MSB:Mamm:41268 | F | 1979 | 36.6025 | -105.486 | 3059 | 0.072831 | 0.461896 | 0.561049 | 35 | 126 | 350 | 7882 | 219 | -141 | 360 | 143 | -71 | 143 | -71 | 443 | 77 | 20 | 51 | 225 | 60 | 225 | 60 |
| *Z. princeps* | ZAPR.SangreCristo | MSB:Mamm:41333 | F | 1979 | 36.6025 | -105.486 | 3059 | 0.076059 | 0.449641 | 0.560064 | 35 | 126 | 350 | 7882 | 219 | -141 | 360 | 143 | -71 | 143 | -71 | 443 | 77 | 20 | 51 | 225 | 60 | 225 | 60 |
| *Z. princeps* | ZAPR.SangreCristo | MSB:Mamm:41334 | M | 1979 | 36.6025 | -105.486 | 3059 | 0.07478 | 0.433048 | 0.526952 | 35 | 126 | 350 | 7882 | 219 | -141 | 360 | 143 | -71 | 143 | -71 | 443 | 77 | 20 | 51 | 225 | 60 | 225 | 60 |
| *Z. princeps* | ZAPR.SangreCristo | MSB:Mamm:41335 | F | 1979 | 36.6025 | -105.486 | 3059 | 0.075834 | 0.446871 | 0.549838 | 35 | 126 | 350 | 7882 | 219 | -141 | 360 | 143 | -71 | 143 | -71 | 443 | 77 | 20 | 51 | 225 | 60 | 225 | 60 |
| *Z. princeps* | ZAPR.SangreCristo | MSB:Mamm:41336 | M | 1979 | 36.6025 | -105.486 | 3059 | 0.076348 | 0.450062 | 0.526832 | 35 | 126 | 350 | 7882 | 219 | -141 | 360 | 143 | -71 | 143 | -71 | 443 | 77 | 20 | 51 | 225 | 60 | 225 | 60 |
| *Z. princeps* | ZAPR.SangreCristo | MSB:Mamm:41337 | F | 1979 | 36.6025 | -105.486 | 3059 | 0.075409 | 0.448531 | 0.555398 | 35 | 126 | 350 | 7882 | 219 | -141 | 360 | 143 | -71 | 143 | -71 | 443 | 77 | 20 | 51 | 225 | 60 | 225 | 60 |
| *Z. princeps* | ZAPR.SangreCristo | MSB:Mamm:41338 | M | 1979 | 36.6025 | -105.486 | 3059 | 0.076231 | 0.443091 | 0.51734 | 35 | 126 | 350 | 7882 | 219 | -141 | 360 | 143 | -71 | 143 | -71 | 443 | 77 | 20 | 51 | 225 | 60 | 225 | 60 |
| *Z. princeps* | ZAPR.SangreCristo | MSB:Mamm:41339 | M | 1979 | 36.6025 | -105.486 | 3059 | 0.077281 | 0.43864 | 0.524307 | 35 | 126 | 350 | 7882 | 219 | -141 | 360 | 143 | -71 | 143 | -71 | 443 | 77 | 20 | 51 | 225 | 60 | 225 | 60 |
| *Z. princeps* | ZAPR.SangreCristo | MSB:Mamm:41340 | M | 1979 | 36.6025 | -105.486 | 3059 | 0.076462 | 0.448953 | 0.532282 | 35 | 126 | 350 | 7882 | 219 | -141 | 360 | 143 | -71 | 143 | -71 | 443 | 77 | 20 | 51 | 225 | 60 | 225 | 60 |
| *Z. princeps* | ZAPR.SangreCristo | MSB:Mamm:41341 | F | 1979 | 36.6025 | -105.486 | 3059 | 0.069627 | 0.378248 | 0.493943 | 35 | 126 | 350 | 7882 | 219 | -141 | 360 | 143 | -71 | 143 | -71 | 443 | 77 | 20 | 51 | 225 | 60 | 225 | 60 |
| *Z. princeps* | ZAPR.SangreCristo | MSB:Mamm:41342 | F | 1979 | 36.6025 | -105.486 | 3059 | 0.07852 | 0.455462 | 0.569526 | 35 | 126 | 350 | 7882 | 219 | -141 | 360 | 143 | -71 | 143 | -71 | 443 | 77 | 20 | 51 | 225 | 60 | 225 | 60 |
| *Z. princeps* | ZAPR.SangreCristo | MSB:Mamm:41343 | M | 1979 | 36.6025 | -105.486 | 3059 | 0.078095 | 0.4389 | 0.537743 | 35 | 126 | 350 | 7882 | 219 | -141 | 360 | 143 | -71 | 143 | -71 | 443 | 77 | 20 | 51 | 225 | 60 | 225 | 60 |
| *Z. princeps* | ZAPR.SangreCristo | MSB:Mamm:41344 | F | 1979 | 36.6025 | -105.486 | 3059 | 0.074182 | 0.386653 | 0.5435 | 35 | 126 | 350 | 7882 | 219 | -141 | 360 | 143 | -71 | 143 | -71 | 443 | 77 | 20 | 51 | 225 | 60 | 225 | 60 |
| *Z. princeps* | ZAPR.SangreCristo | MSB:Mamm:41345 | M | 1979 | 36.6025 | -105.486 | 3059 | 0.076376 | 0.425017 | 0.530733 | 35 | 126 | 350 | 7882 | 219 | -141 | 360 | 143 | -71 | 143 | -71 | 443 | 77 | 20 | 51 | 225 | 60 | 225 | 60 |
| *Z. princeps* | ZAPR.SangreCristo | MSB:Mamm:41346 | F | 1979 | 36.6025 | -105.486 | 3059 | 0.072016 | 0.456643 | 0.53767 | 35 | 126 | 350 | 7882 | 219 | -141 | 360 | 143 | -71 | 143 | -71 | 443 | 77 | 20 | 51 | 225 | 60 | 225 | 60 |
| *Z. princeps* | ZAPR.SangreCristo | MSB:Mamm:41347 | F | 1979 | 36.6025 | -105.486 | 3059 | 0.077489 | 0.460429 | 0.522127 | 35 | 126 | 350 | 7882 | 219 | -141 | 360 | 143 | -71 | 143 | -71 | 443 | 77 | 20 | 51 | 225 | 60 | 225 | 60 |
| *Z. princeps* | ZAPR.SangreCristo | MSB:Mamm:41348 | M | 1979 | 36.6025 | -105.486 | 3059 | 0.076824 | 0.449909 | 0.569118 | 35 | 126 | 350 | 7882 | 219 | -141 | 360 | 143 | -71 | 143 | -71 | 443 | 77 | 20 | 51 | 225 | 60 | 225 | 60 |
| *Z. princeps* | ZAPR.SangreCristo | MSB:Mamm:41349 | M | 1979 | 36.6025 | -105.486 | 3059 | 0.075727 | 0.44663 | 0.55793 | 35 | 126 | 350 | 7882 | 219 | -141 | 360 | 143 | -71 | 143 | -71 | 443 | 77 | 20 | 51 | 225 | 60 | 225 | 60 |
| *Z. princeps* | ZAPR.SangreCristo | MSB:Mamm:41350 | M | 1979 | 36.6025 | -105.486 | 3059 | 0.077995 | 0.422769 | 0.505823 | 35 | 126 | 350 | 7882 | 219 | -141 | 360 | 143 | -71 | 143 | -71 | 443 | 77 | 20 | 51 | 225 | 60 | 225 | 60 |
| *Z. princeps* | ZAPR.SangreCristo | MSB:Mamm:181070 | F | 1974 | 36.7126 | -105.396 | 2736 | 0.073225 | 0.447756 | 0.49767 | 53 | 127 | 349 | 7987 | 238 | -125 | 363 | 161 | -55 | 161 | -55 | 385 | 58 | 20 | 39 | 174 | 61 | 174 | 61 |
| *Z. princeps* | ZAPR.SangreCristo | MSB:Mamm:181071 | F | 1974 | 36.7126 | -105.396 | 2736 | 0.072473 | 0.453946 | 0.515919 | 53 | 127 | 349 | 7987 | 238 | -125 | 363 | 161 | -55 | 161 | -55 | 385 | 58 | 20 | 39 | 174 | 61 | 174 | 61 |
| *Z. princeps* | ZAPR.SangreCristo | MSB:Mamm:270778 | F | 2014 | 36.73292 | -105.483 | 2858 | 0.075526 | 0.378471 | 0.503213 | 45 | 127 | 349 | 7982 | 231 | -134 | 364 | 154 | -51 | 154 | -63 | 429 | 65 | 24 | 39 | 195 | 72 | 195 | 72 |
| *Z. princeps* | ZAPR.SangreCristo | MSB:Mamm:270779 | M | 2014 | 36.73293 | -105.483 | 2858 | 0.075345 | 0.443237 | 0.464828 | 45 | 127 | 349 | 7982 | 231 | -134 | 364 | 154 | -51 | 154 | -63 | 429 | 65 | 24 | 39 | 195 | 72 | 195 | 72 |
| *Z. princeps* | ZAPR.SangreCristo | MSB:Mamm:270777 | M | 2014 | 36.73519 | -105.5 | 2747 | 0.077268 | 0.405327 | 0.520233 | 52 | 127 | 347 | 8035 | 238 | -128 | 366 | 161 | -57 | 161 | -57 | 436 | 66 | 23 | 39 | 198 | 70 | 198 | 70 |
| *Z. princeps* | ZAPR.SangreCristo | MSB:Mamm:270087 | M | 2014 | 36.73555 | -105.5 | 2660 | 0.075728 | 0.410641 | 0.51346 | 57 | 127 | 347 | 8063 | 244 | -123 | 367 | 166 | -52 | 166 | -52 | 427 | 65 | 22 | 40 | 195 | 67 | 195 | 67 |
| *Z. princeps* | ZAPR.CNF | MSB:Mamm:60177 | M | 1976 | 36.8858 | -106.322 | 3204 | 0.076035 | 0.422639 | 0.48637 | 22 | 127 | 346 | 7956 | 210 | -159 | 369 | 132 | 94 | 132 | -86 | 571 | 71 | 26 | 26 | 206 | 89 | 206 | 147 |
| *Z. princeps* | ZAPR.CNF | MSB:Mamm:1600 | F | 1956 | 36.9228 | -106.443 | 3114 | 0.072123 | 0.459379 | 0.52638 | 27 | 127 | 345 | 7968 | 215 | -153 | 368 | -36 | 99 | 137 | -81 | 726 | 84 | 31 | 26 | 240 | 105 | 237 | 205 |
| *Z. princeps* | ZAPR.CNF | MVZ:Mamm:116460 | F | 1952 | 36.99355 | -106.5 | 2802 | 0.075392 | 0.513284 | 0.513092 | 44 | 127 | 342 | 8065 | 234 | -137 | 371 | -19 | 117 | 155 | -66 | 762 | 90 | 33 | 25 | 265 | 112 | 222 | 212 |
| *Z. princeps* | ZAPR.CNF | MVZ:Mamm:116461 | F | 1952 | 36.99355 | -106.5 | 2802 | 0.074138 | 0.488467 | 0.500408 | 44 | 127 | 342 | 8065 | 234 | -137 | 371 | -19 | 117 | 155 | -66 | 762 | 90 | 33 | 25 | 265 | 112 | 222 | 212 |
| *Z. luteus* | ZALU.NENM | DMNS:Mamm:9065 | M | 1997 | 36.99767 | -104.366 | 2358 | 0.077021 | 0.51855 | 0.635769 | 73 | 128 | 357 | 8081 | 261 | -99 | 359 | 183 | -34 | 183 | -34 | 512 | 92 | 14 | 62 | 271 | 43 | 271 | 43 |
| *Z. luteus* | ZALU.NENM | DMNS:Mamm:8630 | M | 1996 | 36.99907 | -104.368 | 2340 | 0.075838 | 0.590626 | 0.602993 | 74 | 128 | 357 | 8087 | 262 | -98 | 360 | 184 | -33 | 184 | -33 | 454 | 84 | 12 | 64 | 248 | 37 | 248 | 37 |
| *Z. luteus* | ZALU.NENM | DMNS:Mamm:8631 | F | 1996 | 37.00037 | -104.361 | 2419 | 0.074079 | 0.556261 | 0.578663 | 69 | 128 | 358 | 8064 | 257 | -102 | 359 | 180 | -38 | 180 | -38 | 531 | 97 | 14 | 63 | 286 | 43 | 286 | 43 |
| *Z. luteus* | ZALU.NENM | DMNS:Mamm:8632 | M | 1996 | 37.00056 | -104.361 | 2419 | 0.075676 | 0.537397 | 0.668937 | 69 | 128 | 358 | 8064 | 257 | -102 | 359 | 180 | -38 | 180 | -38 | 531 | 97 | 14 | 63 | 286 | 43 | 286 | 43 |
| *Z. luteus* | ZALU.NENM | DMNS:Mamm:8633 | F | 1996 | 37.00056 | -104.361 | 2419 | 0.076869 | 0.557721 | 0.687038 | 69 | 128 | 358 | 8064 | 257 | -102 | 359 | 180 | -38 | 180 | -38 | 531 | 97 | 14 | 63 | 286 | 43 | 286 | 43 |
| *Z. luteus* | ZALU.NENM | DMNS:Mamm:8634 | M | 1996 | 37.00056 | -104.361 | 2419 | 0.07721 | 0.526445 | 0.629662 | 69 | 128 | 358 | 8064 | 257 | -102 | 359 | 180 | -38 | 180 | -38 | 531 | 97 | 14 | 63 | 286 | 43 | 286 | 43 |
| *Z. luteus* | ZALU.NENM | DMNS:Mamm:8635 | F | 1996 | 37.00056 | -104.361 | 2419 | 0.077412 | 0.517675 | 0.600795 | 69 | 128 | 358 | 8064 | 257 | -102 | 359 | 180 | -38 | 180 | -38 | 531 | 97 | 14 | 63 | 286 | 43 | 286 | 43 |
| *Z. luteus* | ZALU.NENM | DMNS:Mamm:8636 | M | 1996 | 37.00056 | -104.361 | 2419 | 0.073397 | 0.54524 | 0.597435 | 69 | 128 | 358 | 8064 | 257 | -102 | 359 | 180 | -38 | 180 | -38 | 531 | 97 | 14 | 63 | 286 | 43 | 286 | 43 |
| *Z. luteus* | ZALU.NENM | DMNS:Mamm:8637 | F | 1996 | 37.00056 | -104.361 | 2419 | 0.073642 | 0.546395 | 0.57124 | 69 | 128 | 358 | 8064 | 257 | -102 | 359 | 180 | -38 | 180 | -38 | 531 | 97 | 14 | 63 | 286 | 43 | 286 | 43 |
| *Z. luteus* | ZALU.NENM | DMNS:Mamm:8638 | F | 1996 | 37.00724 | -104.374 | 2358 | 0.078457 | 0.528227 | 0.680241 | 73 | 128 | 357 | 8084 | 261 | -99 | 360 | 183 | -34 | 183 | -34 | 475 | 89 | 12 | 66 | 264 | 37 | 264 | 37 |
| *Z. luteus* | ZALU.NENM | DMNS:Mamm:8639 | F | 1996 | 37.00724 | -104.374 | 2358 | 0.075384 | 0.57838 | 0.643989 | 73 | 128 | 357 | 8084 | 261 | -99 | 360 | 183 | -34 | 183 | -34 | 475 | 89 | 12 | 66 | 264 | 37 | 264 | 37 |
| *Z. luteus* | ZALU.NENM | DMNS:Mamm:8640 | F | 1996 | 37.00724 | -104.374 | 2358 | 0.076585 | 0.611446 | 0.652266 | 73 | 128 | 357 | 8084 | 261 | -99 | 360 | 183 | -34 | 183 | -34 | 475 | 89 | 12 | 66 | 264 | 37 | 264 | 37 |
| *Z. luteus* | ZALU.NENM | DMNS:Mamm:8641 | M | 1996 | 37.00724 | -104.374 | 2358 | 0.075213 | 0.588284 | 0.646069 | 73 | 128 | 357 | 8084 | 261 | -99 | 360 | 183 | -34 | 183 | -34 | 475 | 89 | 12 | 66 | 264 | 37 | 264 | 37 |
| *Z. luteus* | ZALU.DURANGO | MSB:Mamm:10238 | M | 1960 | 37.02711 | -107.49 | 1914 | 0.072078 | 0.47919 | 0.568285 | 98 | 123 | 327 | 8486 | 294 | -81 | 375 | 213 | 179 | 215 | -17 | 310 | 41 | 12 | 26 | 112 | 44 | 72 | 81 |
| *Z. luteus* | ZALU.DURANGO | MSB:Mamm:325322 | F | 2018 | 37.08095 | -107.572 | 1954 | 0.07363 | 0.433822 | 0.524219 | 96 | 122 | 327 | 8466 | 291 | -83 | 374 | 211 | 176 | 212 | -20 | 317 | 42 | 12 | 28 | 115 | 43 | 74 | 83 |
| *Z. luteus* | ZALU.NENM | DMNS:Mamm:21789 | F | 2021 | 37.08119 | -104.472 | 2444 | 0.078052 | 0.54226 | 0.610046 | 67 | 129 | 357 | 8087 | 256 | -105 | 361 | 178 | -40 | 178 | -40 | 675 | 111 | 21 | 53 | 324 | 64 | 324 | 64 |
| *Z. princeps* | ZAPR.NENM | https://arctos.database.museum/guid/DMNS:Mamm:10874 | M | 2003 | 37.19967 | -105.067 | 2824 | 0.084058 | 0.536755 | 0.55936 | 44 | 130 | 354 | 8054 | 233 | -134 | 366 | 154 | -63 | 154 | -63 | 363 | 58 | 16 | 42 | 162 | 50 | 162 | 50 |
| *Z. princeps* | ZAPR.NENM | https://arctos.database.museum/guid/DMNS:Mamm:10875 | F | 2003 | 37.20583 | -105.111 | 3092 | 0.082267 | 0.480425 | 0.560101 | 28 | 130 | 356 | 7975 | 216 | -149 | 365 | 138 | -78 | 138 | -78 | 368 | 58 | 16 | 42 | 162 | 50 | 162 | 50 |
| *Z. luteus* | ZALU.DURANGO | MSB:Mamm:154917 | F | 2007 | 37.2388 | -107.759 | 2088 | 0.072473 | 0.486714 | 0.612369 | 87 | 122 | 328 | 8388 | 282 | -89 | 371 | 201 | 166 | 202 | -27 | 425 | 55 | 16 | 27 | 152 | 56 | 100 | 108 |
| *Z. luteus* | ZALU.DURANGO | MSB:Mamm:155117 | F | 2007 | 37.2388 | -107.759 | 2088 | 0.071472 | 0.460955 | 0.5929 | 87 | 122 | 328 | 8388 | 282 | -89 | 371 | 201 | 166 | 202 | -27 | 425 | 55 | 16 | 27 | 152 | 56 | 100 | 108 |
| *Z. princeps* | ZAPR.NENM | DMNS:Mamm:12891 | M | 2012 | 37.25189 | -105.109 | 3056 | 0.079223 | 0.460268 | 0.550557 | 30 | 130 | 356 | 8010 | 218 | -148 | 367 | 140 | -77 | 140 | -77 | 360 | 54 | 16 | 39 | 154 | 52 | 154 | 52 |
| *Z. princeps* | ZAPR.NENM | DMNS:Mamm:12892 | F | 2012 | 37.25189 | -105.109 | 3056 | 0.078822 | 0.488274 | 0.57408 | 30 | 130 | 356 | 8010 | 218 | -148 | 367 | 140 | -77 | 140 | -77 | 360 | 54 | 16 | 39 | 154 | 52 | 154 | 52 |
| *Z. princeps* | ZAPR.NENM | DMNS:Mamm:7914 | M | 1991 | 37.25302 | -105.11 | 3056 | 0.076779 | 0.486574 | 0.600989 | 30 | 130 | 356 | 8010 | 218 | -148 | 367 | 140 | -77 | 140 | -77 | 360 | 54 | 16 | 39 | 154 | 52 | 154 | 52 |
| *Z. princeps* | ZAPR.NENM | DMNS:Mamm:7915 | F | 1991 | 37.25302 | -105.11 | 3056 | 0.077207 | 0.484931 | 0.578293 | 30 | 130 | 356 | 8010 | 218 | -148 | 367 | 140 | -77 | 140 | -77 | 360 | 54 | 16 | 39 | 154 | 52 | 154 | 52 |
| *Z. princeps* | ZAPR.NENM | DMNS:Mamm:7916 | M | 1991 | 37.25302 | -105.11 | 3056 | 0.077812 | 0.453243 | 0.614504 | 30 | 130 | 356 | 8010 | 218 | -148 | 367 | 140 | -77 | 140 | -77 | 360 | 54 | 16 | 39 | 154 | 52 | 154 | 52 |
| *Z. princeps* | ZAPR.NENM | DMNS:Mamm:7917 | F | 1991 | 37.25302 | -105.11 | 3056 | 0.075213 | 0.474145 | 0.552534 | 30 | 130 | 356 | 8010 | 218 | -148 | 367 | 140 | -77 | 140 | -77 | 360 | 54 | 16 | 39 | 154 | 52 | 154 | 52 |
| *Z. princeps* | ZAPR.NENM | DMNS:Mamm:7918 | F | 1991 | 37.25302 | -105.11 | 3056 | 0.077508 | 0.455289 | 0.567779 | 30 | 130 | 356 | 8010 | 218 | -148 | 367 | 140 | -77 | 140 | -77 | 360 | 54 | 16 | 39 | 154 | 52 | 154 | 52 |
| *Z. princeps* | ZAPR.NENM | DMNS:Mamm:7919 | F | 1991 | 37.25302 | -105.11 | 3056 | 0.076553 | 0.535226 | 0.503903 | 30 | 130 | 356 | 8010 | 218 | -148 | 367 | 140 | -77 | 140 | -77 | 360 | 54 | 16 | 39 | 154 | 52 | 154 | 52 |
| *Z. princeps* | ZAPR.DURANG | MVZ:Mamm:61304 | M | 1933 | 37.3164 | -107.756 | 2450 | 0.072672 | 0.465863 | 0.530042 | 65 | 122 | 333 | 8203 | 259 | -108 | 366 | 178 | 141 | 178 | -46 | 633 | 75 | 25 | 24 | 211 | 87 | 147 | 161 |
| *Z. princeps* | ZAPR.DURANG | MVZ:Mamm:61305 | M | 1933 | 37.3164 | -107.756 | 2450 | 0.072044 | 0.476233 | 0.518398 | 65 | 122 | 333 | 8203 | 259 | -108 | 366 | 178 | 141 | 178 | -46 | 633 | 75 | 25 | 24 | 211 | 87 | 147 | 161 |
| *Z. princeps* | ZAPR.DURANG | MVZ:Mamm:61306 | F | 1933 | 37.3164 | -107.756 | 2450 | 0.073452 | 0.519594 | 0.510225 | 65 | 122 | 333 | 8203 | 259 | -108 | 366 | 178 | 141 | 178 | -46 | 633 | 75 | 25 | 24 | 211 | 87 | 147 | 161 |
| *Z. princeps* | ZAPR.DURANG | MVZ:Mamm:61307 | F | 1933 | 37.3164 | -107.756 | 2450 | 0.071682 | 0.497252 | 0.494192 | 65 | 122 | 333 | 8203 | 259 | -108 | 366 | 178 | 141 | 178 | -46 | 633 | 75 | 25 | 24 | 211 | 87 | 147 | 161 |
| *Z. princeps* | ZAPR.DURANG | MVZ:Mamm:61308 | M | 1933 | 37.3164 | -107.756 | 2450 | 0.072928 | 0.519235 | 0.496255 | 65 | 122 | 333 | 8203 | 259 | -108 | 366 | 178 | 141 | 178 | -46 | 633 | 75 | 25 | 24 | 211 | 87 | 147 | 161 |
| *Z. princeps* | ZAPR.DURANG | MVZ:Mamm:61309 | F | 1933 | 37.3164 | -107.756 | 2450 | 0.07554 | 0.527677 | 0.526185 | 65 | 122 | 333 | 8203 | 259 | -108 | 366 | 178 | 141 | 178 | -46 | 633 | 75 | 25 | 24 | 211 | 87 | 147 | 161 |
| *Z. princeps* | ZAPR.DURANG | MVZ:Mamm:61310 | F | 1933 | 37.3164 | -107.756 | 2450 | 0.075422 | 0.523937 | 0.533794 | 65 | 122 | 333 | 8203 | 259 | -108 | 366 | 178 | 141 | 178 | -46 | 633 | 75 | 25 | 24 | 211 | 87 | 147 | 161 |
| *Z. princeps* | ZAPR.DURANG | MVZ:Mamm:61311 | F | 1933 | 37.3164 | -107.756 | 2450 | 0.072819 | 0.481564 | 0.518333 | 65 | 122 | 333 | 8203 | 259 | -108 | 366 | 178 | 141 | 178 | -46 | 633 | 75 | 25 | 24 | 211 | 87 | 147 | 161 |
| *Z. princeps* | ZAPR.CentralCO | UCM:Mamm:5397 | M | 1952 | 37.80865 | -107.914 | 3190 | 0.078723 | 0.392455 | 0.415485 | 19 | 121 | 339 | 7845 | 210 | -147 | 357 | 129 | 90 | 129 | -85 | 595 | 66 | 32 | 18 | 192 | 107 | 192 | 146 |
| *Z. princeps* | ZAPR.CentralCO | UCM:Mamm:19785 | M | 1993 | 37.82 | -107.28 | 3184 | 0.080865 | 0.342334 | 0.46448 | 17 | 123 | 347 | 7678 | 204 | -151 | 355 | 125 | 84 | 125 | -86 | 380 | 55 | 20 | 33 | 157 | 64 | 157 | 71 |
| *Z. princeps* | ZAPR.CentralCO | UCM:Mamm:19786 | F | 1993 | 37.82 | -107.28 | 3184 | 0.083234 | 0.381766 | 0.469065 | 17 | 123 | 347 | 7678 | 204 | -151 | 355 | 125 | 84 | 125 | -86 | 380 | 55 | 20 | 33 | 157 | 64 | 157 | 71 |
| *Z. princeps* | ZAPR.CentralCO | UCM:Mamm:13741 | F | 1935 | 37.88805 | -108.255 | 3629 | 0.071905 | 0.406462 | 0.459359 | -5 | 120 | 339 | 7784 | 186 | -169 | 355 | 105 | 64 | 105 | -108 | 669 | 83 | 31 | 24 | 241 | 110 | 241 | 149 |
| *Z. princeps* | ZAPR.CentralCO | UCM:Mamm:13737 | M | 1935 | 37.89888 | -107.711 | 3425 | 0.07264 | 0.455708 | 0.457473 | 5 | 121 | 344 | 7685 | 193 | -160 | 353 | 113 | 73 | 113 | -97 | 514 | 62 | 25 | 22 | 179 | 87 | 179 | 115 |
| *Z. princeps* | ZAPR.CentralCO | UCM:Mamm:13738 | F | 1935 | 37.89888 | -107.711 | 3425 | 0.072292 | 0.452614 | 0.433726 | 5 | 121 | 344 | 7685 | 193 | -160 | 353 | 113 | 73 | 113 | -97 | 514 | 62 | 25 | 22 | 179 | 87 | 179 | 115 |
| *Z. princeps* | ZAPR.CentralCO | UCM:Mamm:13739 | M | 1935 | 37.89888 | -107.711 | 3425 | 0.070731 | 0.433713 | 0.470603 | 5 | 121 | 344 | 7685 | 193 | -160 | 353 | 113 | 73 | 113 | -97 | 514 | 62 | 25 | 22 | 179 | 87 | 179 | 115 |
| *Z. princeps* | ZAPR.CentralCO | UCM:Mamm:13740 | F | 1935 | 37.89888 | -107.711 | 3425 | 0.072013 | 0.486345 | 0.433791 | 5 | 121 | 344 | 7685 | 193 | -160 | 353 | 113 | 73 | 113 | -97 | 514 | 62 | 25 | 22 | 179 | 87 | 179 | 115 |
| *Z. princeps* | ZAPR.CentralCO | UCM:Mamm:13742 | F | 1935 | 37.89888 | -107.711 | 3425 | 0.070832 | 0.462932 | 0.433194 | 5 | 121 | 344 | 7685 | 193 | -160 | 353 | 113 | 73 | 113 | -97 | 514 | 62 | 25 | 22 | 179 | 87 | 179 | 115 |
| *Z. princeps* | ZAPR.CentralCO | UCM:Mamm:13743 | M | 1935 | 37.89888 | -107.711 | 3425 | 0.073485 | 0.476878 | 0.479625 | 5 | 121 | 344 | 7685 | 193 | -160 | 353 | 113 | 73 | 113 | -97 | 514 | 62 | 25 | 22 | 179 | 87 | 179 | 115 |
| *Z. princeps* | ZAPR.CentralCO | UCM:Mamm:13744 | M | 1935 | 37.89888 | -107.711 | 3425 | 0.07127 | 0.461721 | 0.445076 | 5 | 121 | 344 | 7685 | 193 | -160 | 353 | 113 | 73 | 113 | -97 | 514 | 62 | 25 | 22 | 179 | 87 | 179 | 115 |
| *Z. princeps* | ZAPR.CentralCO | UCM:Mamm:13745 | M | 1935 | 37.89888 | -107.711 | 3425 | 0.073845 | 0.493969 | 0.477204 | 5 | 121 | 344 | 7685 | 193 | -160 | 353 | 113 | 73 | 113 | -97 | 514 | 62 | 25 | 22 | 179 | 87 | 179 | 115 |
| *Z. princeps* | ZAPR.CentralCO | MSB:Mamm:265966 | M | 2012 | 38.05938 | -105.109 | 2747 | 0.076063 | 0.440866 | 0.536306 | 40 | 138 | 365 | 8149 | 237 | -141 | 377 | 154 | -58 | 154 | -67 | 396 | 58 | 16 | 43 | 168 | 50 | 168 | 51 |
| *Z. princeps* | ZAPR.CentralCO | MSB:Mamm:265991 | M | 2012 | 38.05947 | -105.109 | 2747 | 0.076443 | 0.384347 | 0.534531 | 40 | 138 | 365 | 8149 | 237 | -141 | 377 | 154 | -58 | 154 | -67 | 396 | 58 | 16 | 43 | 168 | 50 | 168 | 51 |
| *Z. princeps* | ZAPR.CentralCO | MSB:Mamm:266947 | M | 2012 | 38.05947 | -105.109 | 2747 | 0.078089 | 0.414677 | 0.525709 | 40 | 138 | 365 | 8149 | 237 | -141 | 377 | 154 | -58 | 154 | -67 | 396 | 58 | 16 | 43 | 168 | 50 | 168 | 51 |
| *Z. princeps* | ZAPR.CentralCO | MVZ:Mamm:61312 | M | 1933 | 38.08304 | -106.498 | 2710 | 0.076743 | 0.458238 | 0.541709 | 39 | 127 | 349 | 7816 | 229 | -134 | 363 | 149 | -51 | 149 | -65 | 245 | 40 | 12 | 42 | 116 | 38 | 116 | 44 |
| *Z. princeps* | ZAPR.CentralCO | MSB:Mamm:124891 | M | 2001 | 38.48406 | -107.188 | 2394 | 0.077311 | 0.349978 | 0.543691 | 56 | 122 | 336 | 7989 | 248 | -115 | 363 | 168 | 127 | 168 | -50 | 306 | 38 | 19 | 21 | 108 | 60 | 108 | 79 |
| *Z. princeps* | ZAPR.CentralCO | MSB:Mamm:101730 | M | 1965 | 38.49902 | -107.451 | 2891 | 0.073129 | 0.461797 | 0.531141 | 30 | 121 | 338 | 7897 | 222 | -137 | 359 | 141 | 99 | 141 | -75 | 441 | 50 | 26 | 18 | 144 | 86 | 144 | 102 |
| *Z. princeps* | ZAPR.CentralCO | MSB:Mamm:124896 | F | 2001 | 38.50397 | -107.024 | 2301 | 0.074474 | 0.403068 | 0.513781 | 60 | 122 | 337 | 7970 | 252 | -112 | 363 | 172 | 0 | 172 | -46 | 203 | 27 | 13 | 25 | 78 | 40 | 78 | 53 |
| *Z. princeps* | ZAPR.CentralCO | MSB:Mamm:103999 | F | 1978 | 38.51678 | -107.036 | 2398 | 0.074351 | 0.402702 | 0.495992 | 54 | 122 | 337 | 7946 | 246 | -116 | 363 | 166 | -5 | 166 | -51 | 253 | 33 | 16 | 25 | 95 | 49 | 95 | 65 |
| *Z. princeps* | ZAPR.CentralCO | MSB:Mamm:104000 | M | 1978 | 38.51678 | -107.036 | 2398 | 0.074349 | 0.408811 | 0.511099 | 54 | 122 | 337 | 7946 | 246 | -116 | 363 | 166 | -5 | 166 | -51 | 253 | 33 | 16 | 25 | 95 | 49 | 95 | 65 |
| *Z. princeps* | ZAPR.CentralCO | MSB:Mamm:104001 | F | 1978 | 38.51678 | -107.036 | 2398 | 0.074256 | 0.41325 | 0.552871 | 54 | 122 | 337 | 7946 | 246 | -116 | 363 | 166 | -5 | 166 | -51 | 253 | 33 | 16 | 25 | 95 | 49 | 95 | 65 |
| *Z. princeps* | ZAPR.CentralCO | MSB:Mamm:124907 | M | 2001 | 38.5308 | -107.313 | 2369 | 0.073914 | 0.389683 | 0.565091 | 57 | 122 | 334 | 8054 | 251 | -113 | 364 | 171 | 129 | 171 | -50 | 219 | 26 | 13 | 19 | 75 | 42 | 75 | 54 |
| *Z. princeps* | ZAPR.CentralCO | UCM:Mamm:7186 | NA | 1955 | 38.54032 | -107.433 | 3058 | 0.07944 | 0.447729 | 0.431558 | 20 | 121 | 340 | 7834 | 211 | -146 | 357 | 131 | 89 | 131 | -84 | 483 | 54 | 28 | 16 | 155 | 93 | 155 | 114 |
| *Z. princeps* | ZAPR.CentralCO | MSB:Mamm:101160 | F | 1968 | 38.55047 | -107.053 | 2496 | 0.074606 | 0.3879 | 0.583244 | 49 | 122 | 338 | 7919 | 240 | -121 | 362 | 160 | 119 | 160 | -56 | 268 | 35 | 17 | 24 | 100 | 53 | 100 | 68 |
| *Z. princeps* | ZAPR.CentralCO | MSB:Mamm:101161 | M | 1968 | 38.55047 | -107.053 | 2496 | 0.072247 | 0.404683 | 0.585227 | 49 | 122 | 338 | 7919 | 240 | -121 | 362 | 160 | 119 | 160 | -56 | 268 | 35 | 17 | 24 | 100 | 53 | 100 | 68 |
| *Z. princeps* | ZAPR.CentralCO | MSB:Mamm:101162 | M | 1968 | 38.55047 | -107.053 | 2496 | 0.071566 | 0.441626 | 0.574823 | 49 | 122 | 338 | 7919 | 240 | -121 | 362 | 160 | 119 | 160 | -56 | 268 | 35 | 17 | 24 | 100 | 53 | 100 | 68 |
| *Z. princeps* | ZAPR.CentralCO | MSB:Mamm:101163 | M | 1968 | 38.55047 | -107.053 | 2496 | 0.073624 | 0.382034 | 0.60646 | 49 | 122 | 338 | 7919 | 240 | -121 | 362 | 160 | 119 | 160 | -56 | 268 | 35 | 17 | 24 | 100 | 53 | 100 | 68 |
| *Z. princeps* | ZAPR.CentralCO | MSB:Mamm:101164 | F | 1968 | 38.55047 | -107.053 | 2496 | 0.073055 | 0.433895 | 0.514695 | 49 | 122 | 338 | 7919 | 240 | -121 | 362 | 160 | 119 | 160 | -56 | 268 | 35 | 17 | 24 | 100 | 53 | 100 | 68 |
| *Z. princeps* | ZAPR.CentralCO | MSB:Mamm:101165 | F | 1968 | 38.55047 | -107.053 | 2496 | 0.079871 | 0.420286 | 0.542708 | 49 | 122 | 338 | 7919 | 240 | -121 | 362 | 160 | 119 | 160 | -56 | 268 | 35 | 17 | 24 | 100 | 53 | 100 | 68 |
| *Z. princeps* | ZAPR.CentralCO | UCM:Mamm:10920 | M | 1903 | 38.86963 | -106.948 | 2845 | 0.077156 | 0.393809 | 0.371713 | 28 | 122 | 340 | 7819 | 218 | -139 | 357 | -31 | 96 | 139 | -75 | 546 | 54 | 28 | 15 | 158 | 94 | 144 | 158 |
| *Z. princeps* | ZAPR.CentralCO | UCM:Mamm:19334 | M | 1970 | 38.88 | -105.15 | 2962 | 0.07794 | 0.401289 | 0.455535 | 21 | 136 | 365 | 7995 | 216 | -158 | 374 | 134 | -84 | 134 | -84 | 643 | 113 | 16 | 61 | 338 | 50 | 338 | 50 |
| *Z. princeps* | ZAPR.CentralCO | UCM:Mamm:19618 | F | 1992 | 38.88 | -105.15 | 2962 | 0.080471 | 0.348602 | 0.395654 | 21 | 136 | 365 | 7995 | 216 | -158 | 374 | 134 | -84 | 134 | -84 | 643 | 113 | 16 | 61 | 338 | 50 | 338 | 50 |
| *Z. princeps* | ZAPR.CentralCO | UCM:Mamm:19635 | M | 1992 | 38.88 | -105.15 | 2962 | 0.080408 | 0.366408 | 0.437411 | 21 | 136 | 365 | 7995 | 216 | -158 | 374 | 134 | -84 | 134 | -84 | 643 | 113 | 16 | 61 | 338 | 50 | 338 | 50 |
| *Z. princeps* | ZAPR.CentralCO | UCM:Mamm:19653 | F | 1992 | 38.88 | -105.15 | 2962 | 0.080881 | 0.395125 | 0.429525 | 21 | 136 | 365 | 7995 | 216 | -158 | 374 | 134 | -84 | 134 | -84 | 643 | 113 | 16 | 61 | 338 | 50 | 338 | 50 |
| *Z. princeps* | ZAPR.CentralCO | UCM:Mamm:19654 | F | 1992 | 38.88 | -105.15 | 2962 | 0.0814 | 0.404351 | 0.456124 | 21 | 136 | 365 | 7995 | 216 | -158 | 374 | 134 | -84 | 134 | -84 | 643 | 113 | 16 | 61 | 338 | 50 | 338 | 50 |
| *Z. princeps* | ZAPR.CentralCO | MVZ:Mamm:154074 | M | 1977 | 38.95916 | -106.989 | 2978 | 0.071895 | 0.379779 | 0.553452 | 20 | 121 | 341 | 7796 | 211 | -146 | 356 | -39 | 87 | 131 | -83 | 474 | 47 | 25 | 14 | 136 | 85 | 117 | 132 |
| *Z. princeps* | ZAPR.CentralCO | MVZ:Mamm:154075 | M | 1977 | 38.95916 | -106.989 | 2978 | 0.074138 | 0.433751 | 0.542004 | 20 | 121 | 341 | 7796 | 211 | -146 | 356 | -39 | 87 | 131 | -83 | 474 | 47 | 25 | 14 | 136 | 85 | 117 | 132 |
| *Z. princeps* | ZAPR.CentralCO | UCM:Mamm:17374 | NA | 1977 | 38.95917 | -106.989 | 2978 | 0.075796 | 0.413986 | 0.401017 | 20 | 121 | 341 | 7796 | 211 | -146 | 356 | -39 | 87 | 131 | -83 | 474 | 47 | 25 | 14 | 136 | 85 | 117 | 132 |
| *Z. princeps* | ZAPR.CentralCO | MVZ:Mamm:125923 | F | 1952 | 38.96278 | -106.989 | 2978 | 0.073839 | 0.507765 | 0.552457 | 20 | 121 | 341 | 7796 | 211 | -146 | 356 | -39 | 87 | 131 | -83 | 474 | 47 | 25 | 14 | 136 | 85 | 117 | 132 |
| *Z. princeps* | ZAPR.CentralCO | MVZ:Mamm:125924 | F | 1952 | 38.96278 | -106.989 | 2978 | 0.072646 | 0.481862 | 0.528127 | 20 | 121 | 341 | 7796 | 211 | -146 | 356 | -39 | 87 | 131 | -83 | 474 | 47 | 25 | 14 | 136 | 85 | 117 | 132 |
| *Z. princeps* | ZAPR.CentralCO | MVZ:Mamm:115551 | F | 1952 | 38.96417 | -106.996 | 2974 | 0.070461 | 0.413216 | 0.512517 | 20 | 121 | 340 | 7800 | 211 | -146 | 356 | -39 | 87 | 131 | -83 | 475 | 47 | 25 | 14 | 137 | 85 | 117 | 132 |
| *Z. princeps* | ZAPR.CentralCO | UCM:Mamm:17376 | M | 1978 | 38.96655 | -106.998 | 2974 | 0.076576 | 0.359013 | 0.420105 | 21 | 121 | 340 | 7806 | 212 | -145 | 357 | -38 | 88 | 132 | -82 | 473 | 47 | 25 | 14 | 137 | 84 | 115 | 132 |
| *Z. princeps* | ZAPR.CentralCO | UCM:Mamm:10919 | M | 1936 | 39.00597 | -107.031 | 3176 | 0.082055 | 0.46558 | 0.47814 | 9 | 121 | 342 | 7744 | 199 | -155 | 355 | -50 | 76 | 120 | -93 | 530 | 53 | 28 | 14 | 153 | 95 | 127 | 145 |
| *Z. princeps* | ZAPR.CentralCO | UCM:Mamm:15142 | F | 1976 | 39.00597 | -107.031 | 3176 | 0.07849 | 0.396188 | 0.448308 | 9 | 121 | 342 | 7744 | 199 | -155 | 355 | -50 | 76 | 120 | -93 | 530 | 53 | 28 | 14 | 153 | 95 | 127 | 145 |
| *Z. princeps* | ZAPR.CentralCO | UCM:Mamm:17375 | M | 1977 | 39.00597 | -107.031 | 3176 | 0.07534 | 0.410415 | 0.418073 | 9 | 121 | 342 | 7744 | 199 | -155 | 355 | -50 | 76 | 120 | -93 | 530 | 53 | 28 | 14 | 153 | 95 | 127 | 145 |
| *Z. princeps* | ZAPR.CentralCO | DMNS:Mamm:12944 | F | 2012 | 39.07567 | -107.953 | 3102 | 0.076348 | 0.39249 | 0.553985 | 17 | 121 | 326 | 8259 | 217 | -153 | 370 | -49 | 89 | 134 | -93 | 386 | 41 | 19 | 16 | 110 | 71 | 93 | 93 |
| *Z. princeps* | ZAPR.CentralCO | DMNS:Mamm:12945 | F | 2012 | 39.07567 | -107.953 | 3102 | 0.078678 | 0.454752 | 0.562209 | 17 | 121 | 326 | 8259 | 217 | -153 | 370 | -49 | 89 | 134 | -93 | 386 | 41 | 19 | 16 | 110 | 71 | 93 | 93 |
| *Z. princeps* | ZAPR.CentralCO | UCM:Mamm:17055 | F | 1993 | 39.19025 | -106.952 | 3049 | 0.081912 | 0.378759 | 0.394629 | 14 | 121 | 340 | 7800 | 206 | -150 | 356 | -11 | 81 | 126 | -88 | 426 | 42 | 24 | 12 | 120 | 81 | 106 | 110 |
| *Z. princeps* | ZAPR.CentralCO | UCM:Mamm:18149 | M | 1993 | 39.19025 | -106.952 | 3049 | 0.081409 | 0.397941 | 0.473156 | 14 | 121 | 340 | 7800 | 206 | -150 | 356 | -11 | 81 | 126 | -88 | 426 | 42 | 24 | 12 | 120 | 81 | 106 | 110 |
